# Supplementary material for: Comprehensive protocol for mixed reality visualization and navigation using 3D Slicer
Source: PLoS One. 2026 Mar 31;21(3):e0343997. doi: 10.1371/journal.pone.0343997 (PMC13038006; doi:10.1371/journal.pone.0343997)
Supplement: S1 File — A step-by-step protocol, also available on protocols.io (https://dx.doi.org/10.17504/protocols.io.kqdg327p1v25/v1). (PDF) [file pone.0343997.s001.pdf]

Oct 27, 2025

Step-by-step protocol for mixed reality visualization and navigation using 3D Slicer

DOI
https://dx.doi.org/10.17504/protocols.io.kqdg327p1v25/v1

Ziyu Qi[1]
[1]Department of Neurosurgery, University of Marburg, Baldingerstrasse, 35043 Marburg, Germany

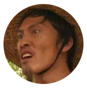

Ziyu Qi
Department of Neurosurgery, University of Marburg, Baldinge...

Create & collaborate more with a free account

Edit and publish protocols, collaborate in communities, share insights through comments, and track progress with run records.

Create free account

OPEN ACCESS

DOI: **https://dx.doi.org/10.17504/protocols.io.kqdg327p1v25/v1**

External link: **https://doi.org/10.6084/m9.figshare.29046527.v1**

Protocol Citation: Ziyu Qi 2025. Step-by-step protocol for mixed reality visualization and navigation using 3D Slicer. **protocols.io** **https://dx.doi.org/10.17504/protocols.io.kqdg327p1v25/v1**

License: This is an open access protocol distributed under the terms of the **Creative Commons Attribution License**, which permits unrestricted use, distribution, and reproduction in any medium, provided the original author and source are credited

Protocol status: In development
We are still developing and optimizing this protocol

Created: July 22, 2024

Last Modified: October 27, 2025

Protocol Integer ID: 103792

Keywords: mixed reality, navigation, segmentation, multimodal imaging, diffusion MRI, surgical planning, 3D Slicer, anonymization, tractography, hologram, guided mixed reality neurosurgical navigation, mixed reality neurosurgical navigation, step protocol for mixed reality visualization, mixed reality visualization, 3d slicer this protocol, 3d slicer, segments within the supplementary demonstration video, using 3d slicer, anatomical segmentation, video segment, linked video segment, multimodal fusion, supplementary demonstration video, relevant video segment, implementing multimodal image, segment, multimodal image, concise troubleshooting guide

Disclaimer

- The authors declare no conflict of interest.
- The funders had no role in the design of the study; in the collection, analyses, or interpretation of data; in the writing of the manuscript; or in the decision to publish the results.
- The demonstration data come from the publicly available MRN dataset (https://doi.org/10.6084/m9.figshare.24550732.v6), which was previously anonymized, and it did not contain any personally identifiable patient information.

Abstract

This protocol provides a comprehensive, step-by-step workflow for implementing multimodal image-guided Mixed Reality Neurosurgical Navigation (MRN) using 3D Slicer. It covers data import, anatomical segmentation, multimodal fusion, surgical planning, and system validation.
It also provides a detailed guide including a comprehensive list of required materials, sequential operational steps, and expected outcomes. Each step is numbered clearly and corresponds directly to segments within the supplementary demonstration videos (available on Figshare https://doi.org/10.6084/m9.figshare.29046527.v9).
Note: Times indicated in parentheses (MM:SS) specify the exact timestamp of the relevant video segment for easy reference. Users are encouraged to follow each step methodically and refer to the linked video segments to enhance understanding and reproducibility.

Protocol structure

```
/
├─Section I: Imaging Data Import
│ └─1 Imaging Data Import
│   ├─1.1 CT Image Import
│   ├─1.2 Structural MRI Import
│   └─1.3 Diffusion MRI Import
├─Section II: Imaging Data Preprocessing
│ ├─2 Data Anonymization
│ │ ├─2.1 CT Data Anonymization
│ │ └─2.2 MRI Data Anonymization
│ ├─3 Imaging Quality Control (QC)
│ │ ├─3.1 CT Data Quality Assessment
│ │ ├─3.2 Structural MRI Quality Assessment
│ │ └─3.3 Diffusion MRI Quality Assessment
│ └─4 Multimodal Image Fusion
│   ├─4.1 Structural MRI Rigid Registration
│   ├─4.2 Diffusion MRI Rigid Registration
│   ├─4.3 Non-Rigid Image Registration
│   └─4.4 Fusion Quality Validation
├─Section III: Anatomical Segmentation and Surgical Planning
│ ├─5 CT Anatomical Segmentation
│ │ ├─5.1 Intracranial Hemorrhage Segmentation
│ │ ├─5.2 Ventricular System Segmentation (CT)
│ │ ├─5.3 Paranasal Sinus Segmentation
│ │ └─5.4 CT Fiducial Marker Segmentation
│ ├─6 Structural MRI Anatomical Segmentation
│ │ ├─6.1 Brain Lesion Segmentation
│ │ ├─6.2 Ventricular System Segmentation (MRI)
│ │ ├─6.3 Venous Vessel Segmentation
│ │ ├─6.4 Arterial Vessel Segmentation
│ │ ├─6.5 Brain Tissue Segmentation
│ │ ├─6.6 Advanced Cerebral Vessel Segmentation
│ │ └─6.7 MRI Fiducial Marker Segmentation
│ ├─7 White Matter Fiber Tractography
│ │ ├─7.1 Whole-Brain Fiber Tractography
│ │ ├─7.2 Motor and Sensory Pathway Segmentation
│ │ └─7.3 Visual Pathway Segmentation
│ ├─8 Surgical Trajectory Planning
│ │ ├─8.1 AC-PC Alignment
│ │ ├─8.2 Key Cranial Landmark Definition
│ │ └─...
│ └─...
└─...

...
│ ├─8.3 Hematoma Aspiration Trajectory
│ ├─8.4 Endoscopic Evacuation Trajectory
│ ├─8.5 Craniotomy and Bone Flap Planning
│ ├─8.6 External Ventricular Drainage (EVD) Planning
│ ├─8.7 Advanced Surgical Path Reconstruction
│ └─8.8 Educational Burr-Hole Skull Model
├─Section IV: Holographic Model Export
│ └─9 3D Model Generation and Export
│   ├─9.1 CT Surface Model Generation
│   ├─9.2 Structural MRI Surface Model Generation
│   ├─9.3 Diffusion MRI Surface Model Generation
│   ├─9.4 CT Holographic Model Export
│   └─9.5 MRI Holographic Model Export
├─Section V: Navigational Registration Support
│ ├─10 Fiducial Marker Localization
│ │ ├─10.1 CT Fiducial Marker Centroid Extraction
│ │ └─10.2 MRI Fiducial Marker Centroid Extraction
│ ├─11 Surface Registration Parameterization
│ │ ├─11.1 Facial Surface Parameterization (CT)
│ │ └─11.2 Facial Surface Parameterization (MRI)
│ └─12 Laser Projection Parameterization
│   ├─12.1 Crosshair Laser Projection (CT)
│   ├─12.2 Crosshair Laser Projection (MRI)
│   ├─12.3 Cone Laser Projection Parameterization
│   └─12.4 Laser Projection Geometry Analysis
├─Section VI: Static Digital Twins Validation
│ ├─13 Physical Validation Model Construction
│ │ ├─13.1 CT Physical Twin Generation
│ │ └─13.2 MRI Physical Twin Generation
│ └─14 Virtual Reference Model Generation
│   ├─14.1 Virtual Reference Planes and Scalp Quadrants (CT)
│   └─14.2 Virtual Reference Planes and Scalp Quadrants (MRI)
└─Section VII: Mixed Reality Navigation Accuracy Assessment
  └─15 Navigational Accuracy and Performance Analysis
    ├─15.1 Accuracy Analysis via Ordered Fiducial Pairs
    ├─15.2 Registration Error Metrics Calculation (FLE and TRE)
    ├─15.3 Extrapolative Error Field and Reliability Analysis
    ├─15.4 Global Registration Error Visualization
    ├─15.5 Local Error Visualization at Anatomical Structures
    ├─15.6 Structure-Specific Registration Accuracy Analysis
    └─15.7 Intrinsic Parameter Computation for Target Structures
```

Protocol structure

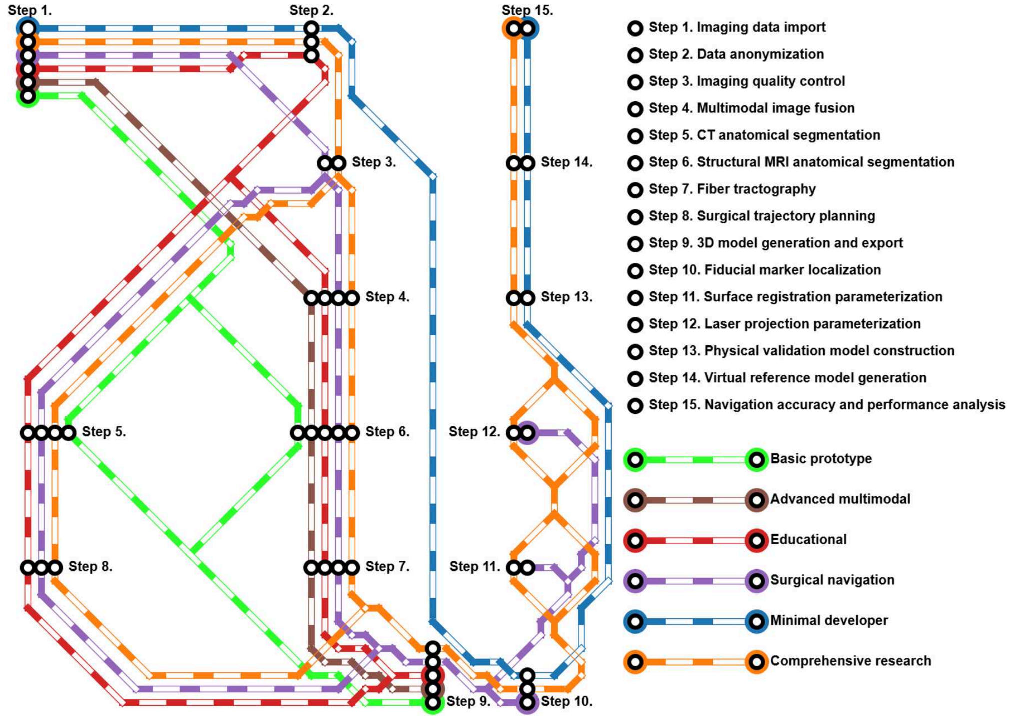

Protocol pipelines

To assist users in quickly addressing potential operational difficulties, a concise troubleshooting guide is provided in the attachment.

Attachments

PDF

S3_Troubleshooting_t...

81KB

Guidelines

- Ensure you use 3D Slicer (version 5.x recommended) with necessary extensions installed (e.g., SlicerDMRI, Elastix).
- It is highly recommended to save your progress regularly, especially immediately after computationally intensive steps, such as whole-brain fiber tractography, to prevent loss from unexpected software crashes.

Materials

Software

- **3D Slicer (version 5.x recommended)**

Required Extensions for 3D Slicer:

To install, open 3D Slicer, navigate to **View → Extension Manager.**

Before you start, ensure that the following modules are **installed** and **enabled**:

- **Add DICOM Data**
- **Compare Volumes**
- **Curve Maker**
- **Data**
- **Diffusion Brain Masking**
- **Diffusion Tensor Estimation**
- **Dynamic Modeler**
- **Export tractography to PLY (mesh)**
- **ExtraMarkups**
- **Fiducials to Model Distance**
- **General Registration (Elastix)**
- **Markups**
- **Merge Models (Deprecated)**
- **MeshStatisticsExtension**
- **Model to Model Distance**
- **Models**
- **Multiply Scalar Volumes**
- **Port Placement**
- **Q3DC**
- **RVXVesselnessFilters**
- **SNR Measurement**
- **Sandbox (Characterize Transform Matrix)**
- **Segment Editor**
- **Segment Statistics**
- **SegmentEditorExtraEffects**
- **Segmentations**
- **SlicerIGT (Fiducial Registration Wizard)**
- **SlicerNeuro (ACPC Transform)**
- **SlicerRT (Segment Comparison)**
- **SlicerVMTK (Extract Centerline)**
- **Subtract Scalar Volumes**
- **Surface Toolbox (requires pyacvd Python package)**
- **Surface Wrap Solidify**
- **Swiss Skull Stripper**
- **Tractography Display**
- **Transforms**

- **UKF Tractography**
- **Volume rendering**
- **Volumes**

Data Requirements
- DICOM format imaging data from clinical MRI (including structural T1, T2, FLAIR, and diffusion MRI sequences) and CT scans.
- Ensure imaging data is ethically anonymized before processing.

Hardware (Recommended for optimal performance)
System requirements (adapted from official 3D Slicer documentation,
https://slicer.readthedocs.io/en/latest/user_guide/getting_started.html#system-requirements):
The protocol runs effectively on modern Windows, macOS, or Linux systems (within the past five years), including virtual machines and Docker containers. Recommended hardware includes at least 8 GB of RAM, a dedicated GPU with OpenGL 3.2 support and sufficient VRAM exceeding dataset size, and a display resolution of 1920 × 1080 pixels or higher. Input devices such as a three-button mouse with a scroll wheel are recommended. An internet connection is also recommended for accessing software extensions, online documentation, datasets, and tutorials.

Troubleshooting

Safety warnings

- Improper anonymization might compromise patient privacy; double-check all datasets before sharing.
- Intensive computational tasks may take significant processing time and potentially cause software instability on insufficient hardware.

Ethics statement

The demonstration data used in this study comes from the MRN dataset publicly available on the Figshare platform (https://doi.org/10.6084/m9.figshare.24550732.v6), licensed under Creative Commons Attribution 4.0 International (CC BY 4.0, **https://creativecommons.org/licenses/by/4.0/**). The MRN dataset was previously anonymized, and no personally identifiable patient information was included. As this research involved only the use of publicly accessible data without identifiable patient information, additional ethics approval and informed consent were not required.

Before start

- Ensure all MRI and CT datasets are correctly anonymized to comply with data privacy and sharing policies.
- Verify that your computer meets the system requirements for running computationally intensive procedures smoothly (at least 16 GB RAM recommended).
- Recommended your computer access to the internet in case of quick support.

Section I: Imaging Data Import

6m 30s

1 **Imaging Data Import**

Note

Modules and Extensions for this step
- **Add DICOM Data**
- **Data**
- **Volumes**

Expected result

Correctly import data and form the foundation for accurate downstream analysis, fusion, segmentation, and navigation procedures.

1.1 **CT Image Import**

2m 28s

Video tutorial at Video_Step_1.1
Actions:
1. Verify that the original DICOM data has been correctly exported from the scanner (00:05–00:18).
2. Open 3D Slicer software and drag the DICOM folder directly into the viewer interface (00:18–00:37).
3. Select "Advanced" mode, carefully inspect and choose the required CT image sequences (e.g., soft tissue window and bone window settings) (00:37–01:14).
4. Review the loaded CT images and associated volume information to confirm correct data import (01:14–01:33).
5. Export anonymized CT image data in the NRRD format, ensuring removal of all patient-identifiable metadata (01:33–02:05).
6. Save the current workspace as an MRB (Medical Reality Bundle) file package for subsequent analysis (02:05–02:33).

Expected result

CT data are successfully imported, anonymized, and exported in NRRD format. Metadata removal is verified, and data are securely saved as an MRB file, ready for further processing.

1.2 **Structural MRI Import** 2m 54s

Video tutorial at Video_Step_1.2

Actions:

1. **Verify** the original structural MRI DICOM files (including T1-weighted, T2-weighted, and FLAIR sequences) have been correctly exported from the scanner (00:05–00:18).
2. **Open** 3D Slicer and **drag and drop** the MRI DICOM folder into the viewer area (00:18–00:36).
3. **Select** "Advanced" mode; carefully **inspect** and **select** the desired MRI sequences, including those acquired on different dates or with various imaging protocols (00:36–00:47).
4. For structural MRI scans (such as T1-weighted sequences), **choose** "Scalar Volume" as the data type and **load** the images into the software (00:47–00:58).
5. **Import** additional structural MRI sequences (contrast-enhanced T1-weighted, T2-weighted, and FLAIR) following the same procedure as above (00:58–02:00).
6. **Rename** each MRI sequence briefly and clearly for easier identification and management (02:00–02:30).
7. **Export** anonymized MRI data in NRRD format, confirming complete removal of patient metadata (02:30–02:59).

Expected result

Structural MRI sequences (T1, T2, FLAIR) are accurately imported, systematically renamed, anonymized, and successfully exported in NRRD format without metadata. Data integrity is confirmed, ensuring readiness for further analysis.

1.3 **Diffusion MRI (dMRI) Import** 1m 8s

Video tutorial at Video_Step_1.3

Actions:

1. Locate the original diffusion MRI (dMRI) sequences within the DICOM database, typically labeled as "diff" or similar terms indicating diffusion imaging (00:05–00:18).
2. Import the diffusion MRI data explicitly by selecting the "DWI Volume" option in the data examination window (00:18–00:25).
3. Verify correct import of diffusion tensor imaging (DTI) data, and clearly rename the sequence for straightforward identification and management (00:25–00:44).
4. Export anonymized diffusion MRI data into NRRD format, ensuring all patient-identifiable metadata is removed (00:44–01:12).

Safety information

Important note to Action-2.:
If not selected correctly in Action-2., subsequent processing steps will not work properly

Expected result

Diffusion MRI data are successfully imported as a DWI volume, clearly renamed, fully anonymized, and securely saved in NRRD format. Data integrity and tensor imaging quality are confirmed, ready for subsequent fiber tractography analyses.

Section II: Imaging Data Preprocessing

12m 43s

2 **Data Anonymization**

(Optional step – perform only if original DICOM contains identifying metadata)

Note

Modules and Extensions for this step
- **Data**

Expected result

This section outlines procedures for anonymizing CT and MRI imaging data within 3D Slicer, ensuring patient privacy and preparing datasets securely for sharing or further research use.

2.1 **CT Data Anonymization**

Video tutorial at Video_Step_2.1

40s

Note

Dependencies for this substep
- go to step #1.1 CT Image Import

Actions:
1. Open the Data module, select the patient dataset, and rename it by replacing identifiable patient information (e.g., patient's name) with an anonymous ID (00:05–00:20).
2. Save the updated scene file in 3D Slicer to apply changes (00:20–00:27).
3. Verify the anonymization by closing and reopening 3D Slicer, then reload the saved scene to ensure that all patient metadata has been successfully removed (00:27–00:45).

Expected result

Patient identifiers are effectively replaced with anonymous IDs. The saved scene file is confirmed fully anonymized, ensuring patient privacy for secure data sharing.

2.2 **MRI Data Anonymization**
Video tutorial at Video_Step_2.2

36s

Note

Dependencies for this substep

- go to step #1.2 Structural MRI Import
- go to step #1.3 Diffusion MRI Import

Actions:

1. Open the Data module, select the MRI dataset of the patient, and rename it by substituting the patient's real name with an anonymous identifier, such as a numeric ID (00:05–00:20).
2. Save the updated 3D Slicer scene file to finalize the changes (00:20–00:41).

Expected result

MRI dataset identifiers are successfully anonymized, and the updated scene file is saved and verified to ensure complete metadata removal, thus securely facilitating subsequent data sharing.

3 **Imaging Quality Control (QC)**

Expected result

Signal-Noise Ratio (SNR) calculation results of imported datasets for quality control.

3.1 **CT Data Quality Assessment**
Video tutorial at Video_Step_3.1

1m 15s

Note

Dependencies for this substep

- go to step #1.1 CT Image Import

Actions:

1. Open the Segment Editor module and create a new segmentation named "SNR Measurement," specifying the CT bone window as the source volume (00:05–00:17).
2. Draw two circular regions (each 80 mm in diameter, approximately 50 mm apart), then export the segmentation as a Labelmap for analysis (00:17–00:57).
3. Switch to the SNRMeasurement module. Set "Volume 1" to the CT bone window and optionally assign "Volume 2" to the CT soft tissue window for comparative measurement (00:57–01:20).

Expected result

Generated Labelmap for SNR calculation, facilitating quantitative quality assessment of CT imaging.

3.2 **Structural MRI Quality Assessment**
Video tutorial at Video_Step_3.2

1m

Note

Dependencies for this substep

- go to step #1.2 Structural MRI Import

Actions:

1. Create a new segmentation, selecting the T1-weighted image (T1WI) as the source volume (00:05–00:21).
2. Open the Segment Editor module, and using a circular brush (80 mm diameter), draw two circular regions approximately 50 mm apart. Then export this segmentation as a Labelmap (00:21–00:47).
3. Switch to the "SNRMeasurement" module. Set "Volume 1" to T1WI and optionally select another structural MRI sequence as "Volume 2" for comparative assessment (00:47–01:06).

Expected result

Labelmap generated for SNR calculation, facilitating quantitative evaluation of structural MRI image quality.

3.3 **Diffusion MRI Quality Assessment**
Video tutorial at Video_Step_3.3

1m 15s

Note

Dependencies for this substep

- go to step #1.3 Diffusion MRI Import

Actions:
Switch to the "Diffusion Brain Masking" module and generate a baseline volume from diffusion MRI data (00:05–00:18).
Open the Segment Editor module and create a new segmentation named "Diffusion SNR," specifying the baseline volume as the source data (00:18–00:40).
Draw two circular regions (each 80 mm diameter, approximately 50 mm apart), and export these regions as a Labelmap (00:40–00:50).
Switch to the "SNRMeasurement" module. Set "Volume 1" to the baseline volume, and optionally assign "Volume 2" to a structural MRI sequence for reference (00:50–01:20).

Expected result

Baseline volume and segmentation successfully created, enabling quantitative assessment of diffusion MRI data quality.

4 **Multimodal Image Fusion**

Note

Modules and Extensions for this step

- **Compare Volumes**
- **Data**
- **Diffusion Brain Masking**
- **General Registration (Elastix)**
- **Segment Editor**
- **Transforms**
- **Volumes**

Expected result

Fuse structural MRI and diffusion MRI sequences, aligning various imaging modalities precisely, enabling integrated anatomical and functional visualization.

4.1 **Structural MRI Rigid Registration**
Video tutorial at Video_Step_4.1

3m 15s

Note

Dependencies for this substep
- go to step #1.2 Structural MRI Import

Actions:
1. Set the "background" and "foreground" images in the slice view windows to the two MRI sequences intended for fusion (00:05–00:27).
2. Inspect the initial alignment between T1-weighted imaging (T1WI) and contrast-enhanced T1-weighted imaging (T1-CE). If misaligned, adjust the colormap settings to better visualize differences and assess alignment quality (00:27–00:40).
3. Open the "General Registration (Elastix)" module. Set T1WI as the fixed image and T1-CE as the moving image, and select "rigid registration" mode (00:40–01:34).
4. After registration, verify alignment by inspecting the transformation matrix labeled "T1-CE to T1WI" in the "Transforms" module (01:34–01:53).
5. Repeat the same procedure to rigidly register additional structural MRI sequences (e.g., T2-weighted or FLAIR) to the common reference image (T1WI). Use distinct colormaps to visually differentiate the registered images clearly (01:53–03:20).

Expected result

Structural MRI sequences (e.g., T1WI and T1-CE) are accurately aligned. Alignment quality is visually confirmed through colormap adjustments and verification of the transformation matrix.

4.2 **Diffusion MRI Rigid Registration**
Video tutorial at Video_Step_4.2

1m 45s

Note

Dependencies for this substep
- go to step #1.2 Structural MRI Import
- go to step #1.3 Diffusion MRI Import

Actions:
1. Preprocess diffusion MRI data using the "Diffusion Brain Masking" module, which generates a scalar baseline volume for fusion with structural MRI (00:05–00:27).
2. Note that preprocessing also generates a brain mask separating brain tissue from external structures (e.g., skull). The mask is not directly used in fusion and can be disregarded at this step (00:27–00:44).
3. Open the "General Registration (Elastix)" module. Set the fixed image to T1-weighted imaging (T1WI), and the moving image to the diffusion baseline volume ("Output

baseline volume"). Choose "rigid registration" and clearly name the resulting transformation matrix (00:44–01:18).

4. After registration, adjust the colormap of the "Output baseline volume" to visually distinguish it from T1WI and confirm fusion quality. Verify the registration matrix labeled "DTI to T1WI" in the "Transforms" module, which specifies the linear transformation aligning diffusion MRI with structural MRI (01:18–01:50).

Expected result

Diffusion MRI baseline volume accurately registered to the structural MRI (T1WI). Alignment quality visually confirmed using colormaps and validated through the computed transformation matrix.

4.3 **Non-Rigid Image Registration**
Video tutorial at Video_Step_4.3

1m 40s

Note

Dependencies for this substep

- go to step #1.2 Structural MRI Import

Actions:

1. Switch to the "Compare Volumes" module, select pre- and post-fusion image sequences for quality evaluation, and confirm synchronized navigation across three orthogonal slice views (axial, sagittal, coronal) (00:05–00:55).
2. Use the "Layer Reveal Cursor" (checkerboard function), either in standard or zoomed mode, to closely inspect and confirm accurate alignment of image textures and anatomical edges (00:55–01:22).

Expected result

Accurate visual assessment of fusion quality, ensuring precise anatomical alignment confirmed through detailed visual inspection.

4.4 **Fusion quality assessment**
Video tutorial at Video_Step_4.4

1m 17s

Note

Dependencies for this substep

- go to step #1.2 Structural MRI Import
- go to step #1.3 Diffusion MRI Import
- go to step #4.1 Structural MRI Rigid Registration
- go to step #4.2 Diffusion MRI Rigid Registration
- go to step #4.3 Non-Rigid Image Registration

Actions:

1. Switch to the "Compare Volumes" module, select pre- and post-fusion image sequences for quality evaluation, and confirm synchronized navigation across three orthogonal slice views (axial, sagittal, coronal) (00:05–00:55).
2. Use the "Layer Reveal Cursor" (checkerboard function), either in standard or zoomed mode, to closely inspect and confirm accurate alignment of image textures and anatomical edges (00:55–01:22).

Expected result

Accurate visual assessment of fusion quality, ensuring precise alignment of anatomical details.

Section III: Anatomical Segmentation and Surgical Planning

2h 5m 50s

5 **CT Anatomical Segmentation**

Note

Modules and Extensions for this step

- **Data**
- **Segment Editor**
- **SegmentEditorExtraEffects**
- **Volumes**
- **Volume rendering**

Expected result

Accurately segment critical anatomical structures from CT images, facilitating detailed anatomical analyses and surgical planning.

5.1 **Intracranial Hemorrhage Segmentation**
Video tutorial at Video_Step_5.1

2m 10s

Note

Dependencies for this substep

- go to step #1.1 CT Image Import

Actions:

1. Rename the imported CT sequences (soft tissue window and bone window) clearly for easier management (00:05–00:24).
2. Navigate to the slice displaying the largest cross-section of the intracranial hemorrhage.
3. In the Segment Editor module, create a new segmentation and add a new segment specifically labeled "Hemorrhage" (00:24–01:05).
4. Apply the "Threshold" tool to segment the hemorrhage using density differences. Set the threshold range to approximately 55–100 HU, capturing the hemorrhage and some unwanted noise or artifacts (01:05–01:39).
5. Use the "Islands" tool to isolate and retain only the primary hemorrhage segment, eliminating extraneous noise fragments (01:39–01:47).
6. Apply the "Smoothing" tool to fill minor holes within the segmented hemorrhage, ensuring precise volume calculations in later analysis steps (01:47–02:15).
   If needed, go to step #15.7 (Volume calculation).

Expected result

Hemorrhage is clearly segmented, isolated from surrounding tissues and artifacts, and smoothed effectively to ensure accurate volumetric analysis.

5.2 **Ventricular System Segmentation (CT)**
Video tutorial at Video_Step_5.2

6m 8s

Note

Dependencies for this substep

- go to step #1.1 CT Image Import

Actions:

1. Open the Segment Editor module, create a new segmentation, and add two segments for distinguishing ventricular and non-ventricular tissues (00:05–00:30).

2. Use the "Draw" tool on an axial slice with clearly visible ventricles. Roughly outline ventricular boundaries to create the initial seed region ("Segment 1") (00:30–01:00).
3. Repeat the rough outlining process on sagittal (01:00–01:13) and coronal slices (01:13–01:30) to better define ventricular regions.
4. Switch to "Segment 2." Similarly use the "Draw" tool to roughly outline non-ventricular areas. Perform this action across axial, coronal, and sagittal views (01:30–02:45).
5. Navigate through additional slices, continuing the rough outlining of ventricular ("Segment 1") and non-ventricular ("Segment 2") seed regions (02:45–04:49).
6. Select the "Grow from Seeds" tool and click "Initialize" to grow the segmentation iteratively. After completion, inspect the resulting 3D model and click "Apply" if satisfactory (04:49–05:19).
7. To enhance segmentation quality due to CT's typically lower soft-tissue contrast, apply "Gaussian Smoothing" with a kernel size of approximately 1 mm (05:19–05:50).
8. Adjust segment names and colors for clear visualization, then save the finalized segmentation data (05:50–06:13).

Expected result

The ventricular system is fully segmented, clearly separated from non-ventricular tissues, and optimized through Gaussian smoothing to improve visual quality.

5.3 **Paranasal Sinus Segmentation**
Video tutorial at Video_Step_5.3

1m 50s

Note

Dependencies for this substep

- go to step #1.1 CT Image Import

Actions:

1. Open the Segment Editor module, create a new segmentation, and add a new segment specifically labeled "Frontal Sinus" (00:05–00:40).
2. Apply the "Threshold" tool to identify sinus regions, leveraging the significantly lower density of air (set threshold < -100 HU). Select "Use for Mask" rather than directly applying this threshold (00:40–01:00).
3. Use the "Scissors" tool in rectangle-fill mode to selectively fill the region around the frontal sinus based on the threshold mask. Then switch to free-form erase mode to remove areas outside the frontal sinus (01:00–01:35).
4. Finally, apply the "Islands" tool to remove smaller unwanted fragments and refine segmentation clarity (01:35–01:55).

Expected result

Frontal sinus is precisely segmented with minimal extraneous structures, optimized for clear visualization and accurate further analysis.

5.4 **CT Fiducial Marker Segmentation**
Video tutorial at Video_Step_5.4

2m 54s

Note

Dependencies for this substep

- go to step #1.1 CT Image Import

Actions:

1. Open the "Volume Rendering" module, select the "CT Bone" preset, and adjust rendering settings to clearly visualize fiducial marker positions (00:05–00:50).
2. In the Segment Editor module, select the "Threshold" tool, and set threshold values greater than 2000 HU to effectively isolate fiducial markers. Choose "Use for Mask" instead of directly applying the threshold (00:50–01:24).
3. With the "Scissors" tool set to circle-fill mode, carefully fill regions around each fiducial marker guided by the threshold mask. If bone or other high-density noise is included, remove these unwanted fragments using free-form erase mode or the "Islands" tool (01:24–02:38).
4. Adjust the segment colors and names clearly, and save the finalized segmentation data (02:38–02:59).

Expected result

CT fiducial markers are clearly segmented and distinguishable, free from surrounding artifacts, accurately identified, and ready for subsequent centroid calculations.

6 **Structural MRI Anatomical Segmentation**

Note

Modules and Extensions for this step

- **Data**
- **Markups**
- **Multiply Scalar Volumes**
- **RVXVesselnessFilters**
- **Segmentations**
- **Segment Editor**
- **SegmentEditorExtraEffects**
- **Subtract Scalar Volumes**
- **Swiss Skull Stripper**
- **Transforms**
- **Volumes**
- **Volume rendering**

Expected result

Perform precise segmentation of key brain structures from structural MRI sequences, crucial for detailed anatomical mapping and lesion identification.

6.1 **Brain Lesion Segmentation**
Video tutorial at Video_Step_6.1

3m 35s

Note

Dependencies for this substep

- go to step #1.2 Structural MRI Import
- go to step #4.3 Non-Rigid Image Registration
- go to step #4.4 Fusion Quality Validation

Actions:

1. Revert the previously applied fusion transform to the contrast-enhanced T1-weighted (T1-CE) MRI, aligning the volume to the orthogonal grid (00:05–00:25).
2. Identify slices displaying the lesion's maximum dimensions across sagittal, coronal, and axial views (00:25–01:00).
3. Using the Markups module, create a Region of Interest (ROI) closely fitting the lesion's maximum extent in all directions (01:00–01:58).
4. Open the Segment Editor module, create a new segmentation and a segment labeled explicitly for lesion pixels (01:58–02:20).

5. Segment the lesion with the "Local Threshold" tool in two phases. Set distinct threshold ranges for enhancing edges and non-enhancing centers. Select enhancing and non-enhancing areas separately using "Ctrl + Left-click" (02:20–03:13).
6. Apply the "Islands" tool to remove isolated pixels or noise, refining the lesion segmentation (03:13–03:40).

Expected result

Accurately segmented lesion boundaries, clearly distinguishing enhancing and non-enhancing areas, refined for precise analysis.

6.2 **Ventricular System Segmentation (MRI)**
Video tutorial at Video_Step_6.2

4m 25s

Note

Dependencies for this substep

- go to step #1.2 Structural MRI Import

Actions:

1. Select an axial slice clearly displaying the ventricular system (00:05–00:24).
2. Open the Segment Editor module, create a new segmentation with two segments: one representing ventricular and another non-ventricular tissue. The T1 contrast-enhanced (T1-CE) MRI is recommended due to superior soft-tissue contrast (00:24–00:42).
3. Ensure orthogonal grid alignment by clicking the alignment warning icon in the module (00:42–00:45).
4. Use the "Level Tracing" tool to roughly outline ventricular boundaries ("Segment 1") in axial, sagittal, and coronal slices (00:45–02:04).
5. Switch to "Segment 2," outline non-ventricular areas similarly, setting "Editable area" to "outside all visible segments" to avoid overlap (02:04–02:45).
6. Repeat this tracing across additional slices as necessary (02:45–03:11).
7. Select "Grow from Seeds," highlight "Segment 1," and initialize segmentation growth. After completion, inspect the 3D segmentation, and if satisfied, click "Apply" (03:11–03:34).
8. If ventricles appear rough, apply "Gaussian Smoothing" (kernel size ~1 mm) to enhance visual quality (03:34–03:55).
9. Adjust segment colors and names clearly, then save the segmentation (03:55–04:30).

Expected result

Accurately segmented ventricular system with clear delineation from surrounding tissues and enhanced visual clarity.

6.3 **Venous Vessel Segmentation**
Video tutorial at Video_Step_6.3

3m 10s

Note

Dependencies for this substep

- go to step #1.2 Structural MRI Import

Actions:

1. Navigate to a sagittal slice clearly showing venous sinuses, typically near the mid-sagittal plane (00:05–00:20).
2. In the Markups module, create an ROI covering the venous sinus area (00:20–00:36).
3. Open the Segment Editor module, create a new segmentation and a dedicated venous vessel segment (00:36–00:59).
4. Use the "Local Threshold" tool to segment main venous sinuses (threshold >500). Select sinus areas using "Ctrl + Left-click" (00:59–01:13).
5. Create a smaller ROI for tributary veins and segment them using a lower threshold (e.g., >480). Select these regions similarly (01:13–02:05).
6. Remove unintended nearby enhanced tissues (e.g., falx cerebri) with the spherical eraser tool in sagittal and coronal views (02:05–02:50).
7. Refine segmentation further with "Islands" or eraser tools (02:50–03:15).

Expected result

Clear segmentation of venous sinuses and tributaries, effectively separated from adjacent structures and visually refined.

6.4 **Arterial Vessel Segmentation**
Video tutorial at Video_Step_6.4

12m 25s

Note

Dependencies for this substep

- go to step #1.2 Structural MRI Import

Actions:

Part I: Internal Carotid Arteries (ICA) Segmentation

1. Locate the approximate axial and coronal slices showing the bilateral internal carotid arteries (ICA) (00:05–00:30).
2. In the **Markups** module, create an ROI named "ICA," covering the region containing the internal carotid arteries (00:30–01:10).
3. In the **Segment Editor** module, create a new "Segmentation" and add one segment specifically dedicated to ICA (01:10–01:30).
4. Open the "Local Threshold" tool, set threshold values (e.g., greater than 800) to identify ICA. Retain segmented pixels using "Ctrl + Left-click," and remove smaller disconnected pixels using the "Islands" tool, preserving only the main ICA structures (01:30–02:05).

Part II: Anterior Cerebral Arteries (ACA) Segmentation

1. In the **Segment Editor** module, create a new segment named "ACA" to represent the anterior cerebral arteries (ACA) (02:05–02:15).
2. In the **Markups** module, create an ROI named "ACA," covering the ACA vascular territory. Identify the anterior longitudinal fissure in coronal and sagittal views to guide ROI placement (02:15–03:10).
3. In the "Local Threshold" tool, set the threshold to values greater than 450. Adjust the minimum diameter (recommended: 0.01mm) and ensure the ROI is set to "ACA." Retain segmented pixels using "Ctrl + Left-click," and use the scissors tool to remove nearby regions (such as the anterior corpus callosum) and the "Islands" tool to remove small disconnected pixels (03:10–04:37).

Part III: Middle Cerebral Artery (MCA) Segmentation

1. In the **Markups** module, create an ROI named "MCA," covering the middle cerebral artery (MCA) territory. Identify the bilateral Sylvian fissures in coronal and axial views for guidance (04:37–05:43).
2. In the **Segment Editor** module, create a new segment named "MCA" specifically for MCA pixels (05:43–05:50).
3. Open the "Local Threshold" tool and set threshold values greater than 450. Adjust the minimum diameter (recommended: 0.01mm) and ensure the ROI is set to "MCA." Use "Ctrl + Left-click" to retain the pixels. Remove unwanted nearby areas (e.g., anterior and middle skull base regions) using the scissors tool and eliminate small disconnected pixels using the "Islands" tool (05:50–07:59).

Part IV: vertebrobasilar artery (VBA) Segmentation

1. In the **Markups** module, create an ROI named "VBA," covering the vertebrobasilar artery (VBA) territory. Locate the prepontine cistern in coronal and sagittal views for placement guidance (07:59–08:30).
2. In the **Segment Editor** module, create a new segment specifically named "VBA" (08:30–08:50).
3. Open the "Local Threshold" tool, set threshold values greater than 600. Adjust the minimum diameter (recommended: 1 mm) and ensure the ROI is set to "VBA." Retain segmented pixels with "Ctrl + Left-click." Remove adjacent regions (such as internal

jugular veins and posterior cranial fossa high-intensity artifacts) using the scissors tool, and refine using the "Islands" tool (08:50–11:02).

Part V: Merging segments into "Arteries"

1. In the **Segment Editor** module, create a new segment named "Arteries" to merge all artery segments (11:02–11:20).
2. Using the "Logical Operators" tool, perform "copy" and "add" operations to merge ICA, ACA, MCA, and VBA segments into the "Arteries" segment. After merging, optionally remove the individual segments (11:20–12:28).

Expected result

Major arterial structures (ICA, ACA, MCA, VBA) are precisely segmented individually, then merged into a comprehensive arterial model, clearly defined and artifact-free.

6.5 **Brain Tissue Segmentation**
Video tutorial at Video_Step_6.5

2m 55s

Note

Dependencies for this substep

- go to step #1.2 Structural MRI Import

Actions:

1. Switch to "Swiss Skull Stripper," download and import required template (00:05–00:30).
2. Convert imported template to label map format ("Volumes" module) (00:30–00:45).
3. Run skull stripping in "Swiss Skull Stripper" (input volume, template, atlas), generate skull-stripped volume (00:45–01:40).
4. Open Segment Editor, use threshold to segment brain tissue and cerebrospinal fluid, refine with "Islands" tool (01:40–03:00).

Expected result

Clear segmentation of brain tissue after peeling the skull tissue.

6.6 **Advanced Cerebral Vessel Segmentation**
Video tutorial at Video_Step_6.6

11m 50s

Note

Dependencies for this substep

- go to step #1.2 Structural MRI Import
- go to step #4.3 Non-Rigid Image Registration
- go to step #4.4 Fusion Quality Validation

Actions:

1. Switch to the **Subtract Scalar Volume** module; subtract the non-enhanced T1 sequence from the enhanced T1 sequence to obtain a silhouette-enhanced volume highlighting vessels and adipose tissue (00:05–00:30).
2. Move to the **Multiply Scalar Volume** module; multiply the silhouette-enhanced volume by itself, creating a squared image volume with significantly enhanced vessel visibility (00:30–01:15).
3. Switch to the **Zhang Vesselness Filter** module; set the skull-stripped brain as a mask, apply filtering to the squared volume, and generate a dedicated vessel-enhanced volume (01:15–02:00).
4. In the **Segment Editor** module, create a new segmentation named "Vessels." Using the vessel-enhanced volume as the source, segment cerebral vessels via thresholding, and refine the results by removing non-vessel dural enhancement using the scissors and islands tools (02:00–04:50).
5. Switch the source volume to the fused T1-CE image. Create a new segment named "Venous vessels," segment the superior sagittal sinus using thresholding, and refine it with scissors and islands tools (04:50–07:30).
6. Create additional segments: "Internal Carotid Arteries" and "Vertebrobasilar Arteries," segment these arteries individually using thresholding, and refine with scissors and islands tools (07:30–11:00).
7. Finally, create a new segment labeled "All Vessels," and merge the previously segmented arterial and venous structures using the logical operators tool (11:00–11:50).

Expected result

Clearly segmented and differentiated arterial and venous cerebral vessels suitable for visualization and further analysis.

6.7 **MRI Fiducial Marker Segmentation** 2m

Video tutorial at Video_Step_6.7

Note

Dependencies for this substep

- go to step #1.2 Structural MRI Import

Actions:

1. Adjust the layout and temporarily close any unnecessary images or models to declutter your workspace (00:05–00:30).
2. Open the "**Volume Rendering**" module. Adjust the slider settings to visually highlight the fiducial markers clearly (00:30–00:50).
3. Navigate to the **Segment Editor** module and create a new segment dedicated to storing the fiducial marker pixels (00:50–01:15).
4. Open the "Threshold" tool. Due to the fiducial markers' high signal intensity, set the threshold value greater than 1000. Select "Use for Mask" instead of directly applying the threshold (01:15–01:30).
5. Use the "Scissors" tool in circle-fill mode to selectively fill regions around each fiducial marker according to the threshold mask. If small areas of bone or other high-density artifacts are unintentionally included, remove them using the "Islands" tool (01:30–02:05).

Expected result

MRI fiducial markers are segmented distinctly, clearly isolated from high-intensity artifacts, ensuring accurate identification and centroid extraction.

7 **White Matter Fiber Tractography**

Note

Modules and Extensions for this step

- **Data**
- **Diffusion Brain Masking**
- **Diffusion Tensor Estimation**
- **Markups**
- **Tractography Display**
- **Transforms**
- **UKF Tractography**

Expected result

Extract and visualize white matter fiber tracts from diffusion MRI data, supporting functional preservation and surgical pathway planning.

7.1 **Whole-Brain Fiber Tractography**
Video tutorial at Video_Step_7.1

17m 30s

Note

Dependencies for this substep

- go to step #1.3 Diffusion MRI Quality Assessment

Actions:

1. Switch to the "**Diffusion Tensor Estimation**" module. Input the previously computed diffusion brain mask, select the "WLS" mode under "Advanced" settings, and click "Apply" to perform whole-brain tensor estimation (00:05–00:42).
2. Set the foreground and background images to the newly estimated tensor volume and the reference T1-weighted imaging (T1WI), respectively. Apply the previously calculated "DTI to T1WI" transform to align the tensor volume correctly, then inspect the results visually (00:42–01:22).
3. Move to the "**UKF Tractography**" module. Set the input tensor data and the output location, leave other settings at their defaults, and click "Apply." Allow approximately 10–15 minutes for the tractography algorithm to generate white matter fiber tracts based on the tensor image (01:22–02:10).
4. After fiber tracts have been correctly transformed into the reference space, use the "**Tractography Display**" module to visualize the tracts more clearly by selecting the "Tubes" display mode (02:10–02:35).

Note

Important note to Action-3.:
It is highly recommended to save the current scene immediately after completing whole-brain fiber tractography, in order to avoid potential loss of progress due to unexpected software crashes.

Expected result

Complete whole-brain fiber tractography visually displayed as clearly delineated fiber bundles.

7.2 **Motor and Sensory Pathway Segmentation**
Video tutorial at Video_Step_7.2

7m 35s

Note

Dependencies for this substep

- go to step #1.3 Diffusion MRI Import
- go to step #7.1 Whole-Brain Fiber Tractography

Actions:

1. Before starting segmentation, revert the fusion transform applied previously to the DTI sequence, ensuring the tensor volume is aligned to the orthogonal grid (00:05–00:40).
2. Open the **Markups** module and create two ROIs essential for corticospinal tract segmentation: one positioned at the cerebral peduncle on the affected side, and the other at the posterior limb of the internal capsule on the same side. Set these ROIs generously to ensure complete coverage. Since the corticospinal tract typically runs vertically, it appears blue-violet in the tensor map, assisting in accurately positioning the ROIs (00:40–03:46).
3. Move to the "**Tractography Display**" module. Set the first ROI at the cerebral peduncle as "Positive" and extract these fibers into a new, separate fiber tract file. Avoid modifying the original whole-brain tractography file directly, as it can still be used to extract other neural pathways. Then, set the second ROI at the internal capsule as "Positive" to further refine the tract selection (03:46–04:42).
4. To eliminate unrelated fibers included within the defined tracts due to the broad initial ROI settings, create additional "Negative ROIs." Place these negative ROIs in anatomical regions unlikely to be traversed by the corticospinal tract, such as temporal and occipital lobes. Additionally, remove any clearly irrelevant or artifact-related fibers to reduce computational load and improve visualization quality (04:42–07:40).

Expected result

A precise segmentation of corticospinal tract fibers, clearly distinguished from unrelated fibers or noise.

7.3 **Visual Pathway Segmentation**
Video tutorial at Video_Step_7.3

4m 50s

Note

Dependencies for this substep

- go to step #1.3 Diffusion MRI Import
- go to step #7.1 Whole-Brain Fiber Tractography

Actions:

1. Open the **Markups** module and create two ROIs essential for optic radiation segmentation: one positioned at the lateral geniculate nucleus (LGN) on the affected side, and another at the superior and inferior lips of the calcarine fissure on the same side. Set these ROIs generously to ensure comprehensive coverage. Since optic radiation fibers generally run anterior-posteriorly, they typically appear yellow-green in tensor maps, aiding accurate ROI placement (00:05–01:40).
2. Switch to the "**Tractography Display**" module. Set your first ROI at the lateral geniculate nucleus (LGN) as "Positive," and extract these fibers into a new, separate fiber tract file. Then, set your second ROI at the superior and inferior lips of the calcarine fissure as "Positive" to further refine fiber selection (01:40–02:42).
3. Since the two-ROI method includes some unrelated fibers, create additional "Negative ROIs" to exclude fibers from anatomically irrelevant areas, such as the frontal lobe, parietal lobe, and cerebellum. Also, remove unrelated fibers explicitly to reduce computational load and enhance visualization quality (02:42–04:55).

Expected result

Clear and accurate segmentation of optic radiation fibers without contamination from unrelated neural fibers.

8 **Surgical Trajectory Planning**

Note

Modules and Extensions for this step

- **Data**
- **Markups**
- **Port Placement**
- **Segmentations**
- **Segment Editor**
- **SegmentEditorExtraEffects**
- **Segment Statistics**
- **SlicerNeuro** **(ACPC Transform)**
- **Volumes**
- **Volume rendering**

Expected result

Define key anatomical landmarks, surgical trajectories, and cranial access points to ensure safe, accurate, and minimally invasive surgical interventions.

8.1 **Anterior commissure-posterior commissure (AC-PC) Alignment**
Video tutorial at Video_Step_8.1

5m

Note

Dependencies for this substep

- go to step #1.1 Imaging Data Import

Actions:

1. Switch to the "**ACPC Transform**" module. Locate the anterior commissure (AC) and posterior commissure (PC) on the axial images, then confirm their positions in the sagittal view (00:05–01:24).
2. Switch to the "Volume Rendering" module, and adjust the view settings for clear visualization. Then, in the **Markups** module, create a point set named "Midline." Mark key midline landmarks, including the nasion (nose root), intersection of the coronal suture and sagittal suture (bregma), and external occipital protuberance (inion) (01:24–03:00).
3. Continuing in axial images, mark additional midline points along the cerebral falx attachment to the skull, adding these points into the "Midline" point set as well (03:00–03:53).
4. Define and name the output transform as "ACPC Transform," and click "Apply." This creates a standardized alignment transform from the original scan position into the standard AC-PC orientation (03:53–04:20).
5. Finally, apply (but do not harden) the **ACPC Transform** to all relevant objects, including images, segmentations, and fiducial markers. This temporarily aligns these objects into the standardized orientation. You can visually confirm this alignment accuracy within the three orthogonal slice views (04:20–05:05).

Expected result

All objects correctly aligned in a standardized AC-PC coordinate system, visually verifiable in three orthogonal views.

8.2 **Key Cranial Landmark Definition (Kaufmann, Kocher, Keen, Frazier points)**
Video tutorial at Video_Step_8.2

15m 24s

Note

Dependencies for this substep

- go to step #1.1 Imaging Data Import
- go to step #8.1 AC-PC Alignment

Actions:

PART I: Kaufmann's Point

1. Open the "Intersection" mode to display the other two orthogonal planes within each 2D slice view. Identify the midsagittal plane using the anterior fontanelle marked in the previous step (00:05–00:52).
2. In the **Markups** module, define a sagittal plane at the midsagittal line. Create two additional parallel sagittal planes shifted 30 mm laterally to both left ("Midline L+30") and right ("Midline R+30") sides using linear transformations (00:52–02:45).
3. Identify the axial plane through the previously marked nasion (nose root) (02:45–02:55).
4. Create an axial plane 50 mm superior to the nasion ("Nasion S+50") using a linear upward transformation (02:55–03:50).
5. In the **Markups** module, mark Kaufmann's points bilaterally where the sagittal and axial planes intersect (03:50–05:06).

PART II: Kocher's Point

1. Using the previously marked anterior fontanelle, identify the coronal plane of the coronal suture ("Coronal Suture") (05:06–05:50).
2. Define another coronal plane located 15 mm anterior to the coronal suture ("Coronal Suture A+15") using a forward linear transformation (05:50–06:10).
3. In the **Markups** module, mark Kocher's points bilaterally at the intersection of these coronal and sagittal planes (06:10–07:10).

PART III: Frazier's Point

1. Identify the axial plane through the previously marked external occipital protuberance (07:10–08:05).
2. Define another axial plane located 60 mm superior to the external occipital protuberance ("Occipital Tuberosity S+60") using an upward linear transformation (08:05–08:55).
3. In the **Markups** module, mark Frazier's points bilaterally at the intersection of these sagittal and axial planes (08:55–09:50).

PART IV: Keen's Point

1. In the "Volume Rendering" module, using the "CT Muscle" preset, locate the axial plane corresponding to the superior margin of the helix (09:50–10:50).
2. Define an axial plane 30 mm superior to this margin ("Superior Helix S+30") using an upward linear transformation (10:50–11:32).
3. Locate the coronal plane through the external auditory canal (11:32–12:00).

4. Define another coronal plane 30 mm posterior to the external auditory canal ("Ear Canal P+30") using a posterior linear transformation (12:00–13:00).
5. In the **Markups** module, mark Keen's points bilaterally at the intersections of these axial and coronal planes (13:00–14:00).

PART V: Reverse ACPC Transform

1. Finally, hide the reference planes to clearly visualize these key cranial landmarks on the skull surface in the 3D viewer (14:00–14:30).
2. Compute the inverse transform of using the "Invert" button in the "Transforms" module and harden the landmark points. (14:30–15:29).

Safety information

Important note to PART V, Action-2.:
These landmark points correspond only to CT images after applying the ACPC transform. To align these landmarks to their natural scanning position, apply and permanently harden the inverse ACPC transform.

Expected result

Key cranial landmarks clearly identified, accurately placed, and aligned with anatomical references.

8.3 **Hematoma Aspiration Trajectory**
Video tutorial at Video_Step_8.3

4m 14s

Note

Dependencies for this substep

- go to step #1.1 CT Image Import
- go to step #5.1 Intracranial Hemorrhage Segmentation
- go to step #8.1 AC-PC Alignment
- go to step #8.2 Key Cranial Landmark Definition

Actions:

1. Hide unrelated objects, leaving only Kaufmann's points visible for clear planning (00:05–00:25).
2. Switch to the **Models** module and adjust the transparency of the hematoma model to clearly visualize internal structures (00:25–00:47).
3. Open the **Segment Statistics** module to calculate the centroid coordinates of the hematoma, and export these coordinates into a table (00:47–01:19).

4. Switch to the **Markups** module and manually enter the calculated hematoma centroid coordinates into a newly created markup named "Hemorrhage Centroid" (01:19–02:01).
5. Since the hematoma is located on the right side, plan a puncture trajectory aligning the right Kaufmann’s point with the hematoma centroid. Create a new segment named "Puncture Path" in the **Segment Editor** module. Use the scissors tool (with "Fill inside" and "Circle" modes) to define a cylindrical puncture path (02:01–03:19).
6. Next, refine the planned path length by opening the "Volume Rendering" module and visualizing the skin surface. Using the scissors tool (with "Erase outside" and "Freeform" modes), preserve only the segment of the path extending from approximately 3 cm outside the skin surface to the distal end of the hematoma (03:19–04:19).

Expected result

A clearly defined cylindrical surgical trajectory precisely connecting Kaufmann’s point to the hematoma centroid.

8.4 **Endoscopic Evacuation Trajectory**
Video tutorial at Video_Step_8.4

1m 52s

Note

Dependencies for this substep

- go to step #1.1 CT Image Import
- go to step #5.1 Intracranial Hemorrhage Segmentation
- go to step #8.1 AC-PC Alignment
- go to step #8.2 Key Cranial Landmark Definition

Actions:

1. Since the hematoma is located on the right side, plan the endoscopic surgical trajectory by aligning the right Kocher’s point with the hematoma centroid (00:05–00:15).
2. In the **Segment Editor** module, create a new segment named "Endoscopic Path" (00:15–00:40).
3. Using the scissors tool in "Fill inside" and "Circle" modes, define a cylindrical trajectory path connecting these two landmarks (00:40–01:05).
4. Next, refine the planned path length. Open the **Volume Rendering** module to visualize the skin surface clearly. Using the scissors tool in "Erase outside" and "Freeform" mode, preserve only the segment of the path extending from approximately 3 cm outside the skin surface to the distal end of the hematoma (01:05–01:57).

Expected result

An accurately planned and visualized endoscopic trajectory from Kocher's point to the hematoma centroid.

8.5 **Craniotomy and Bone Flap Planning**
Video tutorial at Video_Step_8.5

3m 52s

Note

Dependencies for this substep

- go to step #1.1 CT Image Import
- go to step #5.1 Intracranial Hemorrhage Segmentation
- go to step #8.1 AC-PC Alignment
- go to step #8.2 Key Cranial Landmark Definition
- go to step #8.4 Endoscopic Evacuation Trajectory

Actions:

1. Since the hematoma is located on the right side, plan the introducer surgical trajectory by aligning the right Kocher's point with the hematoma centroid (00:05–00:39).
2. In the **Segment Editor** module, create a new segment named "Introducer" (00:47–01:06).
3. Using the scissors tool in "Fill inside" and "Circle" modes, define a cylindrical surgical corridor. Typically, a diameter of approximately 2.5 cm is suitable for introducer placement (01:06–01:30).
4. Next, create another segment named "Skull," and use the "Threshold" tool to segment bone structures by selecting intensity values greater than 200. Use the "Islands" tool to remove small disconnected bone fragments, reducing rendering load (01:30–02:20).
5. Create a third segment named "Bone Flap." Using the "Logical Operators" tool, calculate the intersection between the "Introducer" and "Skull" segments. Further refine this bone flap by using the scissors tool in "Erase outside" and "Freeform" modes to preserve only the region required for the introducer placement (02:20–03:15).
6. Finally, inspect the bone flap and its relationship to the introducer trajectory and skull in the 3D view. Adjust transparency settings as needed for clear visualization and verification (03:15–03:57).

Expected result

Well-defined cranial bone flap segment precisely aligned with the introducer surgical trajectory.

8.6 **External Ventricular Drainage (EVD) Planning**
Video tutorial at Video_Step_8.6

4m 21s

Note

Dependencies for this substep

- go to step #1.1 CT Image Import
- go to step #5.2 Ventricular System Segmentation (CT)
- go to step #8.1 AC-PC Alignment
- go to step #8.2 Key Cranial Landmark Definition

Actions:

1. Switch to the **Models** module and adjust the transparency settings for clear visualization of the ventricular system (00:05–00:21).
2. In the **Markups** module, identify and mark the foramen of Monro clearly in the coronal view (00:21–00:55).
3. In the **Segment Editor** module, create a new segmentation named "EVD Path," with the first segment named "EVD Path-1" (00:55–01:22).
4. Align Kaufmann's point with the marked foramen of Monro. Using the scissors tool (with "Fill inside" and "Circle" modes), create a cylindrical trajectory. Refine the trajectory length using the scissors tool ("Erase outside" and "Freeform" modes) to remove unnecessary segments (01:22–02:10).
5. Create a second segment named "EVD Path-2," aligning Keen's point with the lateral ventricle (02:10–02:40).
6. Again, use the scissors tool ("Fill inside" and "Circle") to define the trajectory. Refine by removing excess path length with the scissors tool ("Erase outside" and "Freeform") (02:40–03:05).
7. Create a third segment named "EVD Path-3," aligning Frazer's point with the lateral ventricle (03:05–03:30).
8. Similarly, define the trajectory with the scissors tool ("Fill inside" and "Circle"), and remove unnecessary trajectory length with "Erase outside" and "Freeform" modes (03:30–03:55).
9. Finally, inspect these planned trajectories within the 3D view, adjusting model transparency as needed for optimal visualization and verification (03:55–04:26).

Expected result

Clearly defined, distinct EVD trajectories precisely aligned with key anatomical landmarks for safe ventricular access.

8.7 **Advanced Surgical Path Reconstruction**
Video tutorial at Video_Step_8.7

3m 23s

Note

Dependencies for this substep

- go to step #1.1 CT Image Import
- go to step #5.1 Intracranial Hemorrhage Segmentation
- go to step #5.2 Ventricular System Segmentation (CT)
- go to step #8.1 AC-PC Alignment
- go to step #8.2 Key Cranial Landmark Definition

Actions:

1. Switch to the **Port Placement** module; select the right Kaufmann point as the entry point and the hematoma centroid as the target. Define the port tool's diameter and length, then click "Aim Tools at Target" to reconstruct the hematoma aspiration path quickly (00:05–00:45).
2. In the **Data** module, verify the dynamic tool model and its aiming transform matrix. Duplicate this model and apply the "harden transform" to fix the tool's spatial position permanently (00:45–01:15).
3. Return to the **Port Placement** module; select the right Kocher point as entry, use the hematoma centroid as the target, set tool diameter and length, then reconstruct the endoscopic path rapidly. Duplicate and apply "harden transform" again (01:15–02:26).
4. Similarly, plan a ventricular puncture. For example, select the left Kaufmann point as entry and the left foramen of Monroe as the target. Set port parameters, then reconstruct and solidify the ventricular puncture trajectory (02:26–03:28).

Expected result

Rapid, precise reconstruction and visualization of surgical paths suitable for hematoma and ventricular punctures

8.8 **Educational Burr-Hole Skull Model**
Video tutorial at Video_Step_8.8

4m 27s

Note

Dependencies for this substep

- go to step #1.1 CT Image Import
- go to step #5.1 Intracranial Hemorrhage Segmentation
- go to step #5.2 Ventricular System Segmentation (CT)
- go to step #8.1 AC-PC Alignment
- go to step #8.2 Key Cranial Landmark Definition

Actions:

1. Open the **Volume Rendering** module, and adjust rendering parameters to make the skull semi-transparent, highlighting the cortical bone contours (00:05–00:25).
2. Create a new segment named Burrhole. Set the scissors tool to Circle centered fill inside and the Editable area to Inside skull. Using the orthogonal 3D views, create cylindrical burr hole fillings centered at each predefined anatomical landmark: Kaufmann's point aimed slightly lateral toward ipsilateral occipital protuberance (00:25–01:30).
3. Keen's point directed toward the contralateral Keen's point (01:30–02:05).
4. Fraiser's point directed toward the ipsilateral medial canthus (02:05–02:35).
5. Kocher's point directed toward the ipsilateral lateral margin of the foramen magnum, with a slightly larger diameter (02:35–03:30).
6. Adjust the scissors tool to Free form erase inside to remove unintended fillings in the skull base (03:30–03:46).
7. Use the Logical operators tool, first Invert the burr hole segment, then apply the Intersect operation with the original skull segment to obtain an educational skull model featuring burr holes (03:46–04:32).

Expected result

A detailed 3D skull model with accurately positioned burr holes, ideal for neurosurgical education and procedural demonstrations.

Section IV: Holographic Model Export

13m 18s

9 **3D Model Generation and Export**

Note

Modules and Extensions for this step

- **Data**
- **Export tractography to PLY (mesh)**
- **Markups**
- **Models**
- **Segmentations**
- **Segment Editor**
- **Tractography Display**
- **Transforms**
- **Volumes**

Expected result

Convert segmented structures and fiber tracts into precise 3D surface mesh models, exporting for holographic visualization and external analysis.

9.1 **CT Surface Model Generation**
Video tutorial at Video_Step_9.1

54s

Note

Dependencies for this substep

- go to step #1.1 CT Image Import
- Any substep in go to step #5 CT Anatomical Segmentation or in go to step #8 Surgical Trajectory Planning

Actions:

1. Open the **Segmentations** module and create a new segmentation named "CT Models" (00:05–00:13).
2. Using the "Copy/move segments" tab, open existing segmentations one by one and copy the segments into the newly created "CT Models" segmentation (00:13–00:35).
3. Set the output type to "**Models**," then click "Export" to convert the segmentation into surface mesh models. You can set the original segmentation visibility to "off" to verify the newly exported models (00:35–00:59).

Expected result

Successfully exported, visually clear 3D surface mesh models from CT-derived segmentation data, ready for further analysis or holographic visualization.

9.2 **Structural MRI Surface Model Generation**
Video tutorial at Video_Step_9.2

2m 20s

Note

Dependencies for this substep

- go to step #1.2 Structural MRI Import
- go to step #1.3 Diffusion MRI Import
- go to step #4.1 Structural MRI Rigid Registration
- go to step #4.2 Diffusion MRI Rigid Registration
- Any substep in go to step #6 Structural MRI Anatomical Segmentation or in go to step #7 White Matter Fiber Tractography

Actions:

1. Open the **Segmentations** module and create a new segmentation named "MRI Models" (00:05–00:19).
2. Using the "Copy/move segments" tab, open each existing segmentation individually and copy the relevant segments into the new "MRI Models" segmentation (00:19–00:58).
3. Set the output type to "Models," then click "Export" to convert these segments into surface mesh models. You can temporarily hide the original segmentation to verify the newly exported models (00:58–01:38).
4. Notice that, except for the fiducial markers, the lesion, ventricular system, venous sinuses, and arteries do not initially align correctly with the reference T1-weighted imaging (T1WI). This misalignment occurs because these segments were originally created from the contrast-enhanced T1-weighted (T1-CE) sequence. ***Therefore, please do apply the previously computed transform ("T1-CE to T1WI") to these models to ensure their correct spatial calibration*** **(01:38–02:25).**

Expected result

Accurately aligned and spatially calibrated MRI-derived surface mesh models, correctly transformed to match the reference T1WI coordinate space.

9.3 **Diffusion MRI Surface Model Generation**
Video tutorial at Video_Step_9.3

2m 29s

Note

Dependencies for this substep

- go to step #1.3 Diffusion MRI Import
- go to step #4.2 Diffusion MRI Rigid Registration
- go to step #7 White Matter Fiber Tractography

Actions:

1. Switch to the "**Export Tractography to PLY (mesh)**" module. From the dropdown menu, select the fiber tracts you wish to export, define the desired tube diameter, and set the export directory. Click "Export" to convert the fiber tracts into surface mesh files in PLY format, which will be saved in the specified directory (00:05–01:14).
2. Re-import the generated PLY files into 3D Slicer to visualize the fiber tract surface meshes. Apply the previously calculated transform "DTI to T1WI" to align the fiber tract models correctly with your reference MRI image. Additionally, further customize and edit the colors and appearance of the models in the **Models** module (01:14–02:34).

Expected result

Accurately aligned diffusion MRI fiber tract surface mesh models, calibrated precisely to the reference MRI space.

9.4 **CT Holographic Model Export**
Video tutorial at Video_Step_9.4

4m 19s

Note

Dependencies for this substep

- go to step #1.1 CT Image Import
- Any substep in go to step #5 CT Anatomical Segmentation or in go to step #8 Surgical Trajectory Planning
- go to step #9.1 Generation of 3D surface models from CT segmentation

Actions:

1. First, export any remaining segmentations (not previously converted) into surface mesh models using the **Segmentations** module (00:05–01:17).
2. Click the "Save" button, choose the OBJ file format for the surface mesh models, and save them to your designated export directory (01:17–02:20).

3. The OBJ file format is universally compatible and allows these models to be opened with standard tools, such as the built-in Windows 3D Viewer, as well as professional software like CloudCompare. CloudCompare, for example, can be used to inspect vertex information, analyze point clouds, and perform further studies (02:20–04:24).

Expected result

Surface mesh models exported as universally compatible OBJ files, ready for visualization and further analysis in standard 3D viewing or professional software.

9.5 **MRI Holographic Model Export**
Video tutorial at Video_Step_9.5

3m 16s

Note

Dependencies for this substep

- go to step #1.2 Structural MRI Import
- go to step #1.3 Diffusion MRI Import
- go to step #4.1 Structural MRI Rigid Registration
- go to step #4.2 Diffusion MRI Rigid Registration
- Any substep in go to step #6 Structural MRI Anatomical Segmentation or in go to step #7 White Matter Fiber Tractography
- go to step #9.2 Structural MRI Surface Model Generation
- go to step #9.3 Diffusion MRI Surface Model Generation

Actions:

1. First, ensure all intended models have been converted into surface mesh models in the **Segmentations** module (00:05–00:26).
2. Click the "Save" button, choose the OBJ file format, and save the surface mesh models to your designated export path (00:26–01:52).
3. The OBJ file format is universally compatible with professional software such as CloudCompare for advanced tasks, including examining vertex data and performing further analyses. Models exported in OBJ format can also be conveniently viewed using Windows' built-in 3D Viewer (01:52–03:16).

Expected result

MRI-derived surface mesh models successfully exported as OBJ files, compatible with standard viewing tools and ready for detailed examination or further processing.

Section V: Navigational Registration Support

45m 52s

10 Spatial localization of specific fiducial markers

Note

Modules and Extensions for this step
- **Data**
- **Markups**
- **Segment Editor**
- **Segment Statistics**

Expected result

Determine accurate centroid coordinates of fiducial markers, enabling precise spatial referencing across imaging modalities and surgical navigation.

10.1 **CT Fiducial Marker Centroid Extraction**
Video tutorial at Video_Step_10.1

3m 10s

Note

Dependencies for this substep
- go to step #1.1 CT Image Import
- go to step #5.4 CT Fiducial Marker Segmentation

Actions:
1. Hide all unrelated markers and models for clarity (00:05–00:30).
2. Switch to the **Segment Editor** module, select the segmentation named "CT Landmarks," and use the "Islands" tool with the "Split islands to segments" option. This will split the currently combined six fiducial markers into six individual segments (00:30–00:51).
3. Switch to the **Segment Statistics** module. Select the "CT Landmarks" segmentation as input, then calculate and export centroid coordinates of the six fiducial markers into a table (00:51–01:25).
4. Move to the **Markups** module and create a new point set. Use the centroid coordinates from the exported table to generate new fiducial points (01:25–02:36).
5. Click "Save," and export this fiducial point set as an ".fcsv" file. Open this exported ".fcsv" file using any text editor to view centroid coordinates directly or import into other software or scripts for further analyses (02:36–03:15).

Expected result

Accurate centroid coordinates of CT-derived fiducial markers clearly extracted, organized into a point set, and saved in ".fcsv" format, ready for further analyses or external software processing.

10.2 **MRI Fiducial Marker Centroid Extraction**
Video tutorial at Video_Step_10.2

3m 10s

Note

Dependencies for this substep

- go to step #1.2 Structural MRI Import
- go to step #6.7 MRI Fiducial Marker Segmentation

Actions:

1. Hide all unrelated markers and models for clarity (00:05–00:30).
2. Switch to the **Segment Editor** module, select the segmentation named "MRI Landmarks," and use the "Islands" tool with the "Split islands to segments" option. This will split the currently combined six fiducial markers into six individual segments (00:30–00:55).
3. Switch to the **Segment Statistics** module. Select the "MRI Landmarks" segmentation as input, then calculate and export centroid coordinates of the six fiducial markers into a table (00:55–01:20).
4. Move to the **Markups** module and create a new point set. Use the centroid coordinates from the exported table to generate new fiducial points (01:20–02:33).
5. Click "Save," and export this fiducial point set as an ".fcsv" file. Open this exported ".fcsv" file using a text editor to directly view centroid coordinates or import into other software or scripts for further analyses (02:33–03:15).

Expected result

Successfully computed and precisely organized centroid coordinates of MRI-derived fiducial markers, exported as a structured ".fcsv" file suitable for subsequent processing, analysis, or integration into other software platforms.

11 **Surface Registration Parameterization**

Note

Modules and Extensions for this step

- **Data**
- **Markups**
- **Segmentations**
- **Segment Editor**
- **SegmentEditorExtraEffects**
- **Surface Wrap Solidify**
- **Surface Toolbox** (requires pyacvd Python package)

Expected result

Generate detailed facial surface point clouds from CT and MRI, crucial for patient-specific visualization, registration, and surface-based analysis.

11.1 **Facial Surface Parameterization (CT)**
Video tutorial at Video_Step_11.1

7m 38s

Note

Dependencies for this substep

- go to step #1.1 CT Image Import

Actions:

1. Switch to the **Segment Editor** module, create a new segment, and use the threshold tool to segment all non-air-density voxels (00:05–00:50).
2. In the 3D orthogonal view, use the scissors tool (Rectangle mode, erase outside) to retain the frontal and upper facial regions used for surface registration. Remove irrelevant markers, nasal tubes, and other artifacts (00:50–01:30).
3. Apply the **Wrap Solidify** tool with default settings to remove minor holes, producing a solid frontal-facial segment (01:30–01:45).
4. Use the Hollow tool to create an external shell of the frontal-facial segment (01:45–02:00).
5. Refine the segment by using scissors and island tools, ensuring only the frontal facial shell remains (02:00–02:45).
6. Move to the **Segmentations** module, rename the segment as "Facial Surface," and export it as a surface mesh model. Finally, use the Surface Toolbox module to clean and resample the mesh, optimizing vertex count and uniformity for easier downstream processing (02:45–03:50).

Expected result

A simplified, uniformly resampled 3D surface mesh model of the frontal-facial region, optimized for accurate surface registration.

*The following is the original method, which is **deprecated** due to its relative complexity and inefficiency.*

Note

Video tutorial at Video_Step_11.1_Deprecated
Deprecated method (for comparison only)

1. Hide all unrelated markers and models for clarity (00:05–00:20).
2. Switch to the **Segmentations** module and create a new segmentation for extracting the facial surface. First, create a segment named "Air" (00:20–00:30).
3. Use the "Threshold" tool to extract air-filled spaces. Intracranial air-filled structures such as sinuses, ear canals, and mastoid air cells will also be included (00:30–01:05).
4. Use a spherical eraser tool of appropriate size to remove unwanted air connections. First, disconnect the nasal air spaces by erasing at the nostril level (01:05–01:35).
5. Use the "Islands" tool to remove remaining isolated air segments within the sinuses (01:35–01:50).
6. Similarly, the spherical eraser tool disconnects air-filled ear canals bilaterally (01:50–02:10).
7. Use the "Islands" tool again to clear air segments within the middle ear (02:10–02:20).
8. Apply the "Logical Operators" tool with the "Invert" function to convert the segmented air spaces into a solid skull model.At this stage, the segment may include extraneous objects like metal supports or catheters, which can be removed with the scissors tool (02:20–03:30).
9. Apply the "Hollow" tool to hollow out the solid skull model, then refine it using scissors to retain only the outer facial shell (03:30–04:40).
10. Switch to the **Segmentations** module and export the segmentation as a model, which can be renamed as "Facial Surface" (04:40–05:10).
11. Export the surface mesh model as a .ply file. The ".ply" file is a universal point cloud format viewable in applications like Windows' built-in 3D Viewer and compatible with professional software such as CloudCompare for advanced processing (05:10–06:41).
12. Optional step: Since the exported model may contain many vertices and triangles, use the **Surface Toolbox** module to perform remeshing. This step produces a lighter, evenly distributed, optimized point cloud and mesh model (06:41–07:43).

11.2 **Facial Surface Parameterization (MRI)** 7m 32s
Video tutorial at Video_Step_11.2

Note

Dependencies for this substep

- go to step #1.2 Structural MRI Import

Actions:

1. Switch to the **Segment Editor** module, set T1WI as the source volume, and use the threshold tool to segment all non-air-density voxels (00:05–00:55).
2. Apply the Smoothing tool to fill small holes, obtaining a solid head segment (00:55–01:35).
3. In the 3D orthogonal view, select the scissors tool (Rectangle mode, erase outside) to retain the frontal and upper facial regions for surface registration, removing irrelevant markers, nasal tubes, and artifacts (01:35–03:00).
4. Use the **Wrap Solidify** tool with default settings to solidify the frontal facial segment further, removing minor residual holes (03:00–04:00).
5. Apply the Hollow tool and then refine the facial shell segment using scissors and island tools to keep only the external surface (04:00–05:04).
6. Move to the **Segmentations** module, export the refined segment as a surface mesh model, then use the Surface Toolbox to clean and uniformly resample the mesh. Verify the vertex and mesh counts in the **Models** module to ensure optimal simplicity and uniformity (04:00–05:04).

Expected result

A simplified, uniformly resampled MRI-derived facial surface mesh model suitable for accurate surface registration.

An earlier version of this method was more complex and has been ***deprecated***.

Note

Video tutorial at Video_Step_11.2_Deprecated
Deprecated method (for comparison only)

1. Hide unrelated markers and models for clarity (00:05–00:30).
2. Switch to the **Segment Editor** module and create a new segmentation for facial surface extraction. Due to its superior soft tissue contrast, select T1-CE as the source volume. First, create a segment labeled "Air" (00:30–00:45).
3. Use the "Threshold" tool to segment air-filled spaces. Intracranial air-filled structures such as sinuses, external auditory canals, and mastoid air cells will be included initially (00:45–00:55).
4. Use the spherical eraser tool to remove unwanted air connections selectively. Begin by disconnecting air-filled external auditory canals (00:55–01:30).
5. Use the "Islands" tool to remove isolated air segments within the middle ear (01:30–01:40).
6. Continue using the spherical eraser tool to remove air connections in other unwanted regions, such as the nasal cavities (01:50–02:10).
7. Again, use the "Islands" tool to clear isolated air segments within the sinuses (02:10–02:15).
8. Apply the "Logical Operators" tool using the "Invert" function to convert the segmented air spaces into a solid head model (02:15–03:05).
9. Next, use the "Hollow" tool to hollow out the model, then refine using the scissors tool to keep only the facial "shell" (03:05–03:40).
10. Switch to the **Segmentations** module and export the segmentation as a model named "Facial Surface" (03:40–04:10).
11. Important: This model is generated from T1-CE images. If used with reference images such as T1WI, apply the previously calculated "T1-CE to T1WI" transform for correct alignment (04:10–04:20).
12. Export the resulting surface mesh as a ".ply" file, a universally compatible point cloud format, viewable via Windows' built-in 3D Viewer or professional software like CloudCompare for further processing (04:20–06:01).
13. Optional step: For improved efficiency, reduce mesh complexity by using the **Surface Toolbox** module to remesh, creating a uniform, lightweight surface point cloud suitable for advanced analyses (06:01–07:37).

12 **Laser Projection Parameterization**

Note

Modules and Extensions for this step

- **Curve Maker**
- **Data**
- **ExtraMarkups**
- **Markups**
- **Segmentations**
- **Segment Editor**
- **SegmentEditorExtraEffects**
- **SlicerVMTK (Extract Centerline)**
- **Transforms**

Expected result

Simulate scanner laser projections to ensure correct patient positioning, enhancing navigation accuracy in surgical procedures.

12.1 **Crosshair Laser Projection (CT)**
Video tutorial at Video_Step_12.1

8m 20s

Note

Dependencies for this substep

- go to step #1.1 CT Image Import

Actions:

1. Open the **Markups** module. Create three planar markups: axial, sagittal, and coronal. Set the normal vectors of these control points precisely at coordinate zero, thus defining three orthogonal reference or "zero" planes (00:05–02:00).
2. Switch to the **Segment Editor** module. Create a new segmentation to store laser projection lines (LPL). Add the first segment, "Coronal LPL" (02:00–02:40).
3. Use the "Threshold" tool to set air-intensity values and choose the "use for masking" option. Switch to the scissors tool with settings "Fill inside" and "Rectangle." Fill along the projected baseline plane in the 2D views, slightly exceeding the line width for clarity (02:40–03:30).
4. Apply the "Hollow" tool to convert the filled region into a hollow shape. Use the scissors tool again ("Erase outside," "Rectangle") to trim and refine this region closer to the skin contour at the baseline plane (03:30–04:07).
5. Use the "Islands" tool to remove unrelated isolated segments. Optionally, use the "Margin" tool to slightly expand the laser segment, enhancing visibility (04:07–04:25).
6. Repeat the above steps on the sagittal and axial images to create respective laser positioning lines in these planes (04:25–07:30).
7. Use the **Volume Rendering** module to quickly verify the relative positions of the simulated laser lines and the head model (07:30–08:00).
8. Export the segments as surface mesh models via the **Segmentations** module for visualization (08:00–08:35).
9. Switch to the **Extract Centerline** module. Using the Coronal Laser Projection Line as an example, click Auto-detect to automatically identify the start and end points of the centerline. Click Apply to generate a parameterized centerline curve (08:35–09:55).
10. Repeat the above step to similarly generate parameterized centerline curves for the Axial and Sagittal projection lines. Note that some segments may represent noise and should be discarded (09:55–12:00).

11. Next, still using the Coronal line as an example, create a point list in the **Markups** module. Visually inspect each segment by toggling visibility, discarding noise segments, and copy the control points from valid segments to the point list (12:00–13:15).
12. Theoretically, the Anterior-Posterior (A-P) coordinates of the coronal points should be zero. However, some points deviate slightly. Correct this by applying a dimensionality reduction linear transform, forcing all points onto the coronal zero-plane by modifying the second row of the identity diagonal matrix from 1 to 0 (13:15–14:00).
13. Switch to the **Curve Maker** module and generate a standardized laser projection line using these corrected points (14:00–14:40).
14. Use the same procedure to obtain standardized Axial and Sagittal laser projection lines and their parameterized control point lists (14:40–21:00).

Expected result

Clearly defined and accurately positioned laser projection lines (coronal, sagittal, axial) aligned precisely with the skin surface model, ready for visualization and validation.

12.2 **Crosshair Laser Projection (MRI)**
Video tutorial at Video_Step_12.2

9m 2s

Note

Dependencies for this substep

- go to step #1.2 Structural MRI Import

Actions:

1. Hide all unrelated models or markers and reorganize your workspace for clarity (00:05–00:30).
2. Switch to the **Transforms** module and apply the previously calculated transform to align the T1-CE sequence with the reference image coordinate system (00:30–01:05).
3. Switch to the **Markups** module. Create three planar markups: axial, sagittal, and coronal, with their normal vectors precisely set to coordinate zero, defining three orthogonal baseline or reference planes (01:05–02:57).
4. Switch to the **Segment Editor** module. Create a new segmentation for storing laser projection lines (LPL), using the T1-CE sequence as the source volume. Add the first segment, "Coronal LPL" (02:57–03:40).
5. Using the "Threshold" tool, set the intensity threshold for air and enable "use for masking." Switch to the scissors tool (settings: "Fill inside," "Rectangle") and fill along the projected baseline plane in the 2D views, with a slightly wider thickness for clear visualization (03:40–04:15).

6. Use the "Islands" tool to retain only the primary segment. Apply the "Hollow" tool to hollow out the segment, and then refine further using the scissors tool (settings: "Erase outside," "Rectangle") to closely match the skin surface at the baseline (04:15–04:47).
7. Optionally, enhance visibility by slightly expanding the segment outward with the "Margin" tool (04:47–05:16).
8. Repeat these steps on the sagittal and axial images to generate respective laser projection lines (05:16–08:36).
9. Then, quickly verify the alignment and relative positions of simulated laser lines and the head model using the "Volume Rendering" module (08:36–09:09).
10. Switch to the **Extract Centerline** module. Using the Coronal Laser Projection Line as an example, click Auto-detect to identify start and end points, then click Apply to generate a parameterized centerline curve (09:09–09:30).
11. Repeat this process for Axial and Sagittal projection lines. Inspect generated curves carefully, discarding segments identified as noise (09:30–11:20).
12. Create a point list for the Coronal Laser Projection Line. Visually examine each segment, remove noise segments, and copy valid control points into the list (11:20–12:10).
13. To correct minor deviations from the coronal zero-plane (A-P coordinates ≠ 0), apply a dimensionality reduction linear transform by changing the second row of the identity diagonal matrix from 1 to 0 (12:10–12:43).
14. Use the **Curve Maker** module with corrected points to create standardized laser projection lines (12:43–13:15).
15. Repeat this workflow for Axial and Sagittal lines, generating standardized laser projection curves and parameterized point lists for each plane (13:15–16:38).

Expected result

Noise-free, standardized, parameterized laser projection curves suitable for precise spatial alignment.

12.3 **Cone Laser Projection Parameterization** 5m 10s

Note

No dependencies

Method 1: Generatrix approximation
Video tutorial at Video_Step_12.3_Method_1
Actions:

1. Open the **Markups** module and create a new point set to draw a generatrix (line) for the conical laser projection. Current parameters define a cone with an apex angle of approximately 53°, making the height equal to the base diameter (00:05–00:30).
2. Switch to the "Curve Maker" module and generate a line model from the defined point set (00:30–01:05).
3. In the **Data** module, clone the generated line model. Use the **Transforms** module to rotate this clone by 180°, creating a mirrored line (01:05–01:27).
4. Switch to the **Merge Models** module. Merge the original and mirrored line models to form two opposite generatrices of the cone, replacing the original model (01:27–01:40).
5. Delete the previously cloned model. Then clone the newly created generatrix model again, and rotate this clone by 90° using the "**Transforms**" module. Merge this new rotated clone with the previous generatrix model to create four equally spaced generatrices (01:40–02:00).
6. Repeat the cloning, rotation, and merging cycle, progressively bisecting the angle between generatrices. Continue until the generatrices form a sufficiently dense cone surface, although still composed of discrete lines (02:00–04:00).
7. Adjust the cone surface's color and transparency to visually simulate laser projection effects. Further transformations or application-specific adjustments can be applied. Finally, the completed cone model can be exported as a universally compatible OBJ file (04:00–05:15).

Expected result

A detailed, visually realistic conical laser projection model with a dense generatrix structure, adjustable for transparency and color, exported in a widely compatible OBJ format.

Method 2: View frustum transformation
Video tutorial at Video_Step_12.3_Method_2
Actions:

1. Set all three orthogonal 2D slice positions to zero (00:05–00:30).
2. Switch to the **Markups** module. Create a cone markup object with three control points, defining a cone with a height and base diameter of 40 mm. Adjust the perspective in the 3D window, aligning the viewpoint along the cone's central axis toward the base (00:30–01:35).
3. Create a new segmentation. Use the scissors tool (Fill inside, Centered circle mode) to generate a voxel-filled cone matching the current viewpoint. If the created cone does not align with the markup-based cone, use the **Transforms** module to adjust; otherwise, no further adjustment is required (01:35–02:40).
4. Apply the Hollow tool to retain only the cone surface, then trim the cone length with the scissors tool (02:40–03:20).

5. To simulate planar projection, create a new segment representing the projection plane using the scissors tool (Fill inside, Centered rectangle mode) (03:20–04:05).
6. Use the Logical Operators module to intersect the cone and the plane, producing a conic intersection line. Expand this line by one voxel using the Margin tool, representing a voxel-based laser projection (not yet parameterized) (04:05–05:00).
7. Convert the conic intersection line to a surface model. Then, use the Extract Centerline module to extract the centerline from this surface model (05:00–06:00).
8. In the **Markups** module, visually inspect extracted centerline curves, remove noise segments, and copy control points of valid segments into a new point list (06:00–07:15).
9. Use the **Curve Maker** module to convert the point list into a refined, smooth, and uniformly parameterized curve model, suitable for advanced analyses or further adjustments (07:15–08:50).

Expected result

A refined, parameterized, smooth laser projection curve for precise and flexible spatial analyses.

12.4 **Laser Projection Geometry Analysis**
Video tutorial at Video_Step_12.4

1m 50s

Note

Dependencies for this substep
- Any substeps in go to step #12 Laser Projection Parameterization

Actions:
1. Create a closed curve based on the control points stored in the Pointlist to analyze the properties of the projected laser curve (00:05–01:10).
2. Under the Measurements tab, select parameters such as curvature and torsion; these metrics are immediately computed and displayed (01:10–01:30).
3. Export the calculated control point data to a spreadsheet for further analysis (01:30–01:50).

Expected result

Immediate visualization and export of laser projection curve parameters, such as curvature and torsion.

Section VI: Static Digital Twins Validation

22m 21s

13 Physical Validation Model Construction

Note

Modules and Extensions for this step

- **Data**
- **Dynamic Modeler**
- **Merge Models** (Deprecated)
- **Segmentations**
- **Segment Editor**
- **SegmentEditorExtraEffects**
- **Surface Wrap Solidify**

Expected result

Construct accurate head phantom models and custom validation setups, supporting rigorous physical and virtual verification of navigation accuracy.

13.1 CT Physical Twin Generation

Video tutorial at Video_Step_13.1

8m 25s

Note

Dependencies for this substep

- go to step #1.1 CT Image Import

Actions:

1. Switch to the **Segment Editor** module, create a new segment, and use the Threshold tool to segment all non-air-density voxels (00:05–00:40).
2. In the orthogonal 3D view, use the Scissors tool (Free-form, Erase Outside mode) to remove irrelevant CT bed components, markers, nasal tubes, and other artifacts (00:40–03:25).
3. Use the **Wrap Solidify** tool with default settings to close small holes, creating a solid frontal facial segment (03:25–03:55).
4. Apply the Hollow tool to generate the outer shell of the frontal facial segment (03:55–04:07).
5. Refine the segment further with the Scissors and Islands tools to ensure only the cranial shell remains (04:07–04:34).
6. Rename the refined segment as Head Phantom, switch to the **Segmentations** module, and export it as a surface mesh model to obtain the basic head phantom (04:34–05:06).

7. Optionally, if spatial markers (e.g., landmarks or laser projection lines) should be integrated, use the **Dynamic Modeler** module with the Append mode to combine these features with the phantom model (05:06–08:30).

Expected result

A refined head phantom model generated from CT data, optionally integrating spatial markers or lines.

*The following is the original method, which is **deprecated** due to its relative complexity and inefficiency.*

Note

Video tutorial at Video_Step_13.1_Deprecated
Deprecated method (for comparison only)

1. Hide unrelated markers and models for a clearer workspace (00:05–00:30).
2. Switch to the **Segment Editor** module and create a new segmentation for generating head phantom models. Create the first segment named "Air" (00:30–00:50).
3. Use the "Threshold" tool to segment air spaces. Initially, internal air spaces such as sinuses, external auditory canals, and mastoid air cells will be included (00:50–01:11).
4. Select the spherical eraser tool to remove unnecessary air connections. Begin by disconnecting air-filled external auditory canals (01:11–01:25).
5. Use the "Islands" tool to remove air within the nasal sinuses (01:25–01:40).
6. Again, use the spherical eraser to disconnect the air connections at the nasal openings (01:40–01:55).
7. Use the "Islands" tool to remove air segments within the middle ear (01:55–02:06).
8. Apply the "Logical Operators" tool with the "Invert" function, converting segmented air spaces into a solid skull model. At this stage, additional unwanted elements, such as metal backplates, catheters, or marker holders, can be removed using the "Islands" or scissors tool (02:06–05:24).
9. Use the "Hollow" tool to hollow out the skull model, then refine using the scissors tool to retain only the facial "shell" (05:24–05:40).
10. Switch to the **Segmentations** module and export the segmentation as a model, naming it "Head Phantom" (05:40–06:05).
11. Optionally, the **Merge Models** module combines the anatomical head phantom model with known artificial markers (such as fiducial points or lines). This enables the creation of custom head phantoms suitable for various system validation scenarios (06:05–10:32).

13.2 **MRI Physical Twin Generation**
Video tutorial at Video_Step_13.2

5m 37s

Note

Dependencies for this substep

- go to step #1.2 Structural MRI Import

Actions:

1. Switch to the **Segment Editor** module, select T1WI as the source volume, create a new segment, and use the Threshold tool to segment all non-air-density voxels (00:05–00:40).
2. In the orthogonal 3D views, use the Scissors tool (Free-form, Erase Outside mode) to remove irrelevant markers and artifacts (00:40–02:15).
3. Use the Wrap Solidify tool with default settings to fill small holes, creating a solid frontal facial segment (02:15–02:50).
4. Apply the Hollow tool to generate the outer shell, and further refine the shell with Scissors and Islands tools, retaining only the cranial surface (02:50–03:20).
5. Rename this segment as Head Phantom, switch to the **Segmentations** module, and export it as a surface mesh model to obtain the basic head phantom (03:20–03:50).
6. Optionally, if integration with known spatial markers (e.g., landmarks or laser projection lines) is required, use the **Dynamic Modeler** module in Append mode to combine these markers with the phantom model (03:50–05:47).

Expected result

A refined head phantom model generated from T1-weighted MRI, optionally integrated with spatial markers.

An earlier version of this method was more complex and has been ***deprecated***.

Note

Deprecated method (for comparison only)
Video tutorial at Video_Step_13.2_Deprecated
Actions:

1. Hide unrelated markers and models, switch to the T1-CE sequence, and apply the transform aligning it with the reference image coordinate system (00:05–00:55).
2. Switch to the **Segment Editor** module and create a new segmentation specifically for generating head phantom models, selecting T1-CE as the source volume. Add the first segment named "Air" (00:55–01:20).
3. Use the "Threshold" tool to segment air spaces. Initially, internal structures such as sinuses, external auditory canals, and mastoid air cells will also be included (01:20–01:44).
4. Select the spherical eraser tool to remove unwanted air connections. First, disconnect air-filled external auditory canals (01:44–01:52).
5. Continue by using the spherical eraser tool to disconnect nasal air connections at the nostrils (01:52–02:20).
6. Use the "Islands" tool to remove isolated air segments within nasal sinuses and middle ears (02:20–02:33).
7. Rename the "Air" segment as "Head Phantom." Apply the "Logical Operators" tool with the "Invert" function, converting the segmented air spaces into a solid head model (02:33–02:50).
8. Use the "Hollow" tool to hollow out the solid head model, then refine it further with the scissors tool to retain only the facial "shell" (02:50–03:15).
9. Switch to the **Segmentations** module and export the refined segmentation as a model (03:15–03:34).
10. Optionally, use the **Merge Models** module to combine this anatomical head phantom with known artificial markers (e.g., fiducial points or fiducial lines), creating customized phantom models for various system validation purposes (03:34–06:44).

14 Virtual Reference Model Generation

Note

Modules and Extensions for this step

- **Data**
- **Dynamic Modeler**
- **Markups**
- **Merge Models (Deprecated)**
- **Segmentations**
- **Segment Editor**
- **SegmentEditorExtraEffects**

Expected result

Create virtual anatomical reference planes and segmented scalp quadrants for precise validation of surgical planning and navigation outcomes.

14.1 **Virtual Reference Planes and Scalp Quadrants (CT)**
Video tutorial at Video_Step_14.1

4m 24s

Note

Dependencies for this substep

- go to step #1.1 CT Image Import
- go to step #13.1 CT Physical Twin Generation

Concept of scalp quadrants (SQs): The scalp surface divided into four regions aiming to simplify evaluation.

Actions:

1. Switch to the **Segment Editor** module and create a new segment named "Basic Planes" (00:05–00:25).
2. Set the slice positions in each 2D view to zero (0) to define reference planes (00:25–00:38).
3. Using the Scissors tool (Rectangle mode, Fill inside), fill along the baseline planes (yellow lines) in each 2D view (00:40–01:55).
4. Switch to the **Segmentations** module and export these three reference planes as surface mesh models (01:55–02:10).
5. To create scalp quadrant models, switch to the **Dynamic Modeler** module. In Plane Cut mode, use the axial, coronal, and sagittal planes to cut the Head Phantom model orthogonally, creating eight spatial quadrants. Typically, retain two diagonally opposite scalp quadrants on the top as alignment references. Use Append mode to merge these quadrants (02:10–05:10).
6. Adjust the color and transparency settings to visualize scalp quadrants effectively as virtual alignment references (05:10–06:01).

Expected result

Orthogonal reference planes and selected scalp quadrant models generated, serving as virtual references for alignment and validation.

*The following is the original method, which is **deprecated** due to its relative complexity and inefficiency.*

Note

Deprecated method (for comparison only)
Video tutorial at Video_Step_14.1_Deprecated
Actions:

1. In the **Segment Editor** module, create a new segmentation named "Validating Objects" for representing validation models. Begin by adding one segment to store pixels representing the virtual reference planes (00:05–00:30).
2. Switch to the **Markups** module and make the three previously defined planar markups clearly visible in the viewing windows (00:30–00:51).
3. Return to the **Segment Editor** module and carefully use the scissors tool (with settings: "Fill inside," "Rectangle") to fill along the projections of each of the three markup-defined planes in the 2D views. After completion, verify these filled segments of the three baseline planes in the 3D view (00:51–01:42).
4. Switch to the **Segmentations** module. Copy the "Head Phantom" segment into the "Validating Objects" segmentation (01:42–02:04).
5. Return to the **Segment Editor** module and, using orthogonal 3D views, preserve two opposite quadrants of the upper head model, selected according to your specific surgical planning needs. For example, you might retain the left anterior quadrant and the right posterior quadrant (02:04–03:20).
6. Switch to the **Segmentations** module, export these selected quadrants as mesh models ("scalp quadrants") for validation purposes (03:20–04:29).

14.2 **Virtual Reference Planes and Scalp Quadrants (MRI)**
Video tutorial at Video_Step_14.2

3m 55s

Note

Dependencies for this substep

- go to step #1.2 Structural MRI Import
- go to step #13.2 MRI Physical Twin Generation

Concept of scalp quadrants (SQs): The scalp surface divided into four regions aiming to simplify evaluation.

Actions:

1. Set each 2D view orientation to axial, coronal, and sagittal and adjust slice positions to zero (0). Ensure that reformatted views are reset to standard orientations (00:05–00:30).
2. Switch to the **Segment Editor** module, select T1WI as the source volume, and create a new segment named "Basic Planes" (00:30–00:40).
3. Use the Scissors tool (Rectangle mode, Fill inside) to fill along the baseline planes (highlighted in yellow) in each 2D view (00:40–01:40).
4. Export these three baseline planes as surface mesh models through the Segmentations module (01:40–02:12).
5. Switch to the **Dynamic Modeler** module and use Plane Cut mode to orthogonally divide the Head Phantom into eight spatial quadrants, using the axial, coronal, and

sagittal planes. Typically, two diagonally opposite scalp quadrants on the top are retained and merged using the Append mode for alignment reference purposes (02:12–05:20).

6. Adjust color and transparency settings to effectively visualize the scalp quadrants as virtual alignment references (05:20–06:11).

Expected result

Orthogonal baseline planes and selected scalp quadrant models are clearly generated, providing precise virtual references for spatial alignment and validation.

An earlier version of this method was more complex and has been ***deprecated***.

Note

Video tutorial at Video_Step_14.2_Deprecated
Deprecated method (for comparison only)

1. Hide unrelated models and markers to clear your workspace (00:05–00:22).
2. Switch to the **Markups** module and make the three previously defined planar markups clearly visible in the viewing windows (00:22–00:30).
3. In the **Segment Editor** module, create a new segmentation named "Validating Objects." Add one segment specifically for the baseline reference planes (00:30–01:00).
4. Use the scissors tool (settings: "Fill inside," "Rectangle") to carefully fill along the three markup-defined planes in the 2D views. Confirm that these filled segments representing baseline planes are correctly visualized in the 3D view (01:00–01:30).
5. Switch to the **Segmentations** module. Copy the "Head Phantom" segment into the "Validating Objects" segmentation and rename this segment as "Scalp Quadrant" (01:30–02:14).
6. Switch back to the **Segment Editor**. Using orthogonal 3D views, preserve two opposite quadrants of the upper head model based on your specific surgical scenario. For example, retain the left anterior and right posterior quadrants (02:14–03:20).
7. Optionally, adjust transparency or colors to visually inspect the positional relationships between virtual reference planes, scalp quadrants, and other existing mesh models (03:20–04:00).

Section VII: Mixed Reality Navigation Accuracy Assessment

32m 10s

15 **Navigational Accuracy and Performance Analysis**

Note

Modules and Extensions for this step

- **Data**
- **Fiducials to Model Distance**
- **Markups**
- **Merge Models (Deprecated)**
- **MeshStatisticsExtension**
- **Models**
- **Model to Model Distance**
- **Q3DC**
- **Sandbox (Characterize Transform Matrix)**
- **Segmentations**
- **Segment Editor**
- **SegmentEditorExtraEffects**
- **Segment Statistics**
- **SlicerIGT (Fiducial Registration Wizard)**
- **SlicerRT (Segment Comparison)**
- **Transforms**

Expected result

Quantitatively evaluate MR navigation system accuracy, employing rigorous metrics, error field visualization, and target-specific analyses for comprehensive quality assurance.

15.1 **Accuracy Analysis via Ordered Fiducial Pairs**
Video tutorial at Video_Step_15.1

4m 33s

Note

Dependencies for this substep

- go to step #1 Imaging Data Import
- go to step #10 Fiducial Marker Localization

Actions:

1. Drag and drop the point-pair files intended for comparison directly into 3D Slicer (00:05–00:15).
2. In **Markups** module, or instance, differentiate clearly among the centroid sets from fiducial markers—such as actual centroid set "***C***," user-observed virtual marker set "***V***," and physical marker set "***P***"—by adjusting their color settings (00:15–00:55).
3. Rename individual point labels within each set for simplified and clear visualization (00:55–02:15).

4. In the **Markups** module, click to create a "Closed Curve." Copy all points from a specific set—for example, set "***P***"—into the control points of the closed curve labeled "***P***-Set." Set the curve type to "Linear" to visually connect these points, forming a polygon (02:15–03:32).
5. Repeat this procedure to create closed polygons for sets "***V***" and "***C***" as well (03:32–04:15).
6. Visually assess accuracy by examining deviations between corresponding points or line segments, facilitating evaluation of the matching accuracy between specific points or overall sets (04:15–04:38).

Expected result

Clear visualization of fiducial marker sets ("***C***," "***V***," and "***P***") represented as closed polygons, allowing straightforward visual evaluation of matching accuracy between these sets.

15.2 **Registration Error Metrics Calculation (*FLE* and *TRE*)**
Video tutorial at Video_Step_15.2

1m 42s

Note

Dependencies for this substep

- go to step #1 Imaging Data Import
- go to step #10 Fiducial Marker Localization
- go to step #15.1 Accuracy Analysis via Ordered Fiducial Pairs

Actions:

1. Open the **Fiducials to Model Distance** module. Select two ordered fiducial point sets intended for comparison—for example, the virtual markers (***V***) and the actual centroid set (***C***). Specify an output table, then click "Apply". The resulting table displays individual Fiducial Localization Error (FLE_i), representing point-to-point deviations, along with the overall ***FLE*** for the entire set (00:05–01:06).
2. Repeat this process by selecting the physical marker set (***P***) and the centroid set (***C***), and again specify an output table. Click "Apply". Similarly, this generates a table displaying individual Target Registration Errors (TRE_i), showing point-to-point deviations, along with the overall ***TRE*** for the entire set (01:06–01:47).
3. Switch to the **Q3DC** module and select the Calculate Distance Between Two Landmarks tab; enter the corresponding point pairs individually. The module outputs directional displacement components in RL, AP, and SI orientations (i.e., TRE_{ix}, TRE_{iy}, TRE_{iz}, FLE_{ix}, FLE_{iy}, and FLE_{iz}), as well as Euclidean distances consistent with prior calculations (01:47–03:10).

4. Copy and integrate these newly computed results into the existing spreadsheet for consolidated analysis (03:10–04:28).

Expected result

Accurately computed ***FLE*** and ***TRE***, clearly tabulated and ready for quantitative evaluation of navigation accuracy. Detailed directional (RL, AP, SI) and Euclidean displacement data obtained and integrated with existing measurements.

15.3 **Extrapolative Error Field and Reliability Analysis**
Video tutorial at Video_Step_15.3

5m 17s

Note

Dependencies for this substep

- go to step #1 Imaging Data Import
- go to step #10 Fiducial Marker Localization
- go to step #15.1 Accuracy Analysis via Ordered Fiducial Pairs

Actions:

1. Clone the fiducial centroid "***C***-Set," and rename this cloned set as "***P∗***-Set" for subsequent transformations (00:05–00:20).
2. Switch to the **Fiducial Registration Wizard** module. Compute the registration transform from set "***C***" to set "***P***," labeled as T_C^P. This transform represents the extrapolated error field or extrapolation transform. Apply this transform to the "***P∗***-Set," creating an extrapolated physical point set (00:20–01:15).
3. Change the color of the "***P∗***-Set" points to differentiate clearly from existing point sets. Create a closed polygon from this extrapolated point set for better visual inspection and analysis (01:15–02:30).
4. Switch to the "Fiducials to Model Distance" module. Compare the "***P∗***-Set" with the actual "***P***-Set," calculating individual Fiducial Registration Errors (FRE_i) and the overall Fiducial Registration Error (***FRE***) (02:30–03:20).
5. Export the T_C^P matrix as a ".mat" file, suitable for further analysis in MATLAB. Upon loading into MATLAB, you will see a 4×4 homogeneous transformation matrix. The first three rows include rotational and translational components. Given the rigid registration mode, the fourth row of the matrix can be completed as [0 0 0 1]. Further MATLAB scripts can compute additional quality metrics from this matrix, such as the Frobenius norm, to analyze the reliability of the extrapolation (03:20–05:22).
6. Switch to the **Fiducial Registration Wizard** module, register ***C***-Set to ***V***-Set to compute the user-perceived offset field, labeled as T_C^V (05:22–05:59).

7. Create a duplicate of ***C***-Set, then apply the transform T_C^V to this duplicate, resulting in an extrapolated virtual fiducial set named "***V****-Set" (05:59–07:28).
8. Switch to the Characterize Transform Matrix module to inspect and interpret the geometric properties of the offset fields T_C^P and T_C^V (07:28–08:08).

Here is a MATLAB code to calculate the Frobenius Norm (FN) of T_C^P and T_C^V

```
%% FN of Error Matrix T_C_P and T_C_V
[FN_TCP FN_TCV] = norm([T_C_P T_C_V] - eye(4), 'fro');
```

Expected result

Extrapolated physical fiducial points ("***P****-Set") and extrapolated virtual fiducial points ("**V***-Set") generated using computed registration transforms (T_C^P and T_C^V), clearly visualized and quantitatively analyzed (***FRE***), with exported transformation matrix ready for advanced MATLAB analysis.

15.4 Global visualization of registration error fields

4m 52s

Video tutorial at **Video_Step_15.4**

Note

Dependencies for this substep

- go to step #1 Imaging Data Import
- go to step #10 Fiducial Marker Localization
- go to step #15.1 Accuracy Analysis via Ordered Fiducial Pairs
- go to step #15.3 Extrapolative Error Field and Reliability Analysis

Actions:

1. Switch to the "**Transforms**" module. Under the "Display" tab, visualize the registration error field on specific 2D slices using "Glyph," "Grid," or "Contour" display modes. Error magnitudes are represented using a color scale (00:05–01:20).
2. To analyze the properties of the error field in 3D space, use the "Convert" tab to export a scalar Volume representing the error field, aligned with the reference image coordinate system. Each voxel in this Volume corresponds geometrically to the reference image, and the voxel intensity indicates the magnitude of error at that point. Using the "Volume" module, adjust the window width and level threshold settings to evaluate the spatial distribution of specific ranges of error magnitude (01:20–02:30).

3. Since the error field volume is scalar, it can be segmented. For example, create a segmentation to visualize different error ranges in increments of 2 mm, generating distinct error-level regions. Because the transformation is rigid, these segmented error ranges typically appear as coaxial cylinders. The axis of these cylinders represents the geometric direction of principal error eigenvectors (02:30–04:57).

Expected result

Comprehensive global visualization of registration errors as scalar volumes, providing intuitive insight into spatial error distribution through color-coded visualization and segmentation of distinct error magnitude ranges.

15.5 **Local Error Visualization at Anatomical Structures**
Video tutorial at Video_Step_15.5

6m 41s

Note

Dependencies for this substep

- go to step #1 Imaging Data Import
- go to step #10 Fiducial Marker Localization
- go to step #15.1 Accuracy Analysis via Ordered Fiducial Pairs
- go to step #15.3 Extrapolative Error Field and Reliability Analysis

Actions:

1. Hide unrelated models and markers, and create copies of the target models intended for evaluation (00:05–01:00).
2. Open the **Transforms** module and apply the extrapolated error field transform to these model copies. This simulates the spatial mismatch between the virtual models and their physical counterparts in the 3D view (01:00–01:29).
3. Switch to the **Model to Model Distance** module. Set the original model as the source model and the transformed copy as the "Target Model." Select the option "Corresponding point-to-point distance," and choose the transformed copy as the output model. Click "Apply." Repeat this operation for each pair of models being evaluated (01:29–02:45).
4. Switch to the **Models** module. In the "Scalars" tab, apply a Colormap to visualize displacement magnitudes on each evaluated model. To ensure consistency in color scaling across models, manually set the range to 0–10 mm. In the 3D view, lower displacement appears greenish, while higher displacement is represented by reddish hues. This facilitates direct visual assessment of registration errors at specific anatomical targets (02:45–06:46).
5. Switch to the **Fiducials to Model Distance** module, select the models containing the computed error scalar maps, check the desired statistical fields, and click Run

(06:46–07:25).

6. Results will appear in the tab below and can be exported as a CSV file for viewing or further analysis in Excel (07:25–08:12).

Expected result

Detailed visual comparison of transformed and original models, with color-mapped displacement magnitudes clearly highlighting areas of highest registration discrepancy, facilitating precise assessment of local accuracy;
Statistical parameters of the error scalar maps are calculated and exported for detailed numerical analysis.

15.6 **Structure-Specific Registration Accuracy Analysis**
Video tutorial at Video_Step_15.6

3m 2s

Note

Dependencies for this substep

- go to step #1 Imaging Data Import
- Any substeps in go to step #9 3D Model Generation and Export
- go to step #10 Fiducial Marker Localization
- go to step #15.1 Accuracy Analysis via Ordered Fiducial Pairs
- go to step #15.3 Extrapolative Error Field and Reliability Analysis

Actions:

1. Hide unrelated models and markers to simplify the workspace (00:05–00:20).
2. Duplicate the "Lesion" segmentation, creating a copy, and apply the extrapolated error field transform to this copied segmentation (00:20–00:50).
3. Move both the original and transformed segments into the same directory for direct comparison (00:50–02:05).
4. Adjust colors and transparency settings of both segments to visually assess their overlap and discrepancy. This visual mismatch effectively represents the registration error between virtual and physical structures (02:05–02:20).
5. Open the "**Segment Comparison**" module. Quantitatively compare the two segments to calculate accuracy metrics such as Hausdorff distance and Dice coefficient (02:20–03:07).

Expected result

Clear visual and quantitative evaluation of segmentation accuracy, facilitating understanding of registration errors.

15.7 **Intrinsic Parameter Computation for Target Structures**
Video tutorial at Video_Step_15.7

6m 3s

Note

Dependencies for this substep

- go to step #1 Imaging Data Import
- Any substeps in go to step #5 CT Anatomical Segmentation or go to step #6 Structural MRI Anatomical Segmentation
- go to step #10 Fiducial Marker Localization
- go to step #15.1 Accuracy Analysis via Ordered Fiducial Pairs
- go to step #15.3 Extrapolative Error Field and Reliability Analysis

Actions:Actions:

1. Calculate intrinsic parameters (centroid, volume) using Segment Statistics module (00:05–00:45).
2. Define fiducial point ("Lesion Centroid") and measure Euclidean distance to origin (01:20–02:24).
3. Compute **distance** from lesion centroid to nearest skin surface point using "Model to **Model** Distance," visualize using Markups lines (02:24–05:32).
4. Inspect visually to verify measured distances (05:32–06:08).

Expected result

Validated centroid location and intrinsic lesion parameters with precise distance measurements from anatomical landmarks.

Acknowledgements

We would like to express our sincere appreciation to Dr. Hui Zhang for her invaluable assistance during drafting this protocol.
