# Supplementary material for: Comprehensive protocol for mixed reality visualization and navigation using 3D Slicer
Source: PLoS One. 2026 Mar 31;21(3):e0343997. doi: 10.1371/journal.pone.0343997 (PMC13038006; doi:10.1371/journal.pone.0343997)
Supplement: S3 Table — Listed are common problems encountered in each step, possible underlying causes, and recommended corrective actions to ensure smooth protocol execution. (PDF) [file pone.0343997.s003.pdf]

Table S3. Troubleshooting Guide

Step	Problem	Possible Cause	Recommended Solution
1. Imaging Data Import	**a)** Failure to import DICOM files into 3D Slicer.	**a)** DICOM dataset incomplete or corrupted; unsupported file format.	**a)** Ensure no special characters (e.g., Chinese) in the path. Verify that all DICOM files are present and not corrupted. If drag-and-drop fails, use Slicer's DICOM module to import (ensuring the DICOM database is set up). Check that the data format is supported (convert proprietary formats if needed).
	**b)** The wrong image series or orientation was loaded.	**b)** The scanner exported multiple series (e.g., localizers or duplicates), and the wrong one was selected, or there was a misinterpretation of file names.	**b)** Double-check series descriptions in the DICOM browser (look for correct sequence name, e.g., "T1-weighted" vs "localizer"). Load images via the DICOM module, which groups series properly. Confirm orientation by scrolling through slices – if anatomy looks incorrect (e.g., left-right flipped), ensure proper DICOM orientation flags or re-import with corrected metadata.
	**c)** Software memory error or crash on import	**c)** Very large imaging files (e.g., high-resolution diffusion data) can exceed memory or trigger a bug in an older Slicer version.	**c)** Use a 64-bit system with sufficient RAM for large datasets; import one modality at a time rather than all at once. For diffusion MRI, ensure the DICOM includes gradient tables – if a crash persists, convert DICOM to NRRD using Slicer's Diffusion Import tool to reduce overhead. Save the scene after each import to avoid redoing work if a crash occurs.
2. Data Anonymization	**a)** Patient identifiers still visible after "anonymization"	**a)** Not all metadata was removed (e.g., DICOM tags in hidden fields), or the user saved in the original format without clearing the header.	**a)** Use Slicer's anonymization routine and export to NRRD or NIfTI, which strips personal DICOM tags. Double-check by inspecting image metadata (File → Information) to ensure no IDs remain. If using an external tool, confirm that fields like Patient Name, ID, etc., are blank.
	**b)** Data not saved or exported correctly.	**b)** The user might have forgotten to save the processed files in the new format or saved only the scene file (.mrml) without the data.	**b)** Always use "Save As" and choose NRRD/NIfTI for images after anonymization. Ensure that, along with the scene file, the volume files themselves are saved (check the output directory for .nrrd files). If using the MRB package, verify that it contains the new volumes.
	**c)** Volume appears distorted or wrong after conversion	**c)** Voxel dimensions or orientation could change if not handled properly during export (especially between DICOM and NRRD).	**c)** Verify image geometry before vs. after anonymization – check slice spacing and orientation. Use Slicer's Data module to compare original vs. anonymized volume. If a discrepancy is found, ensure the correct reference was used (e.g., apply any needed transforms before saving).
3. Imaging Quality Control (QC)	**a)** Unrealistic or "NA" SNR values are displayed.	**a)** The "SNR measurement" segmentation ROI might be placed in an inappropriate region (e.g., air or bone)	**a)** Ensure that the ROI for SNR includes a uniform region of tissue and an adjacent background region. For example, measure signal in a homogenous area (like white matter or a phantom region) and noise in the air.
	**b)** The SNR value seems low.	**b)** It may be an issue with ROI selection, or the image itself is of low quality.	**b)** If user error is ruled out, the image itself likely has inherently low SNR. Avoid drastic interpolation in subsequent segmentations (Steps 5 and 6).
	**c)** Unclear SNR measurement procedure.	**c)** Users might not understand how to execute the SNR measurement	**c)** Treat this step as an optional quality assessment. Emphasize that skipping it does not affect subsequent steps, but it is useful for checking image quality.
4. Multimodal Image Fusion	**a)** Misaligned images after registration.	**a)** Wrong fixed/moving image assignment or incorrect initial positioning. For example, if T1 and T2 were not roughly aligned before running Elastix, the algorithm might have converged on the wrong local minimum.	**a)** Always set the higher-quality or anatomical reference as the fixed image (e.g., use T1 as fixed, register other modalities to it). Before fine registration, perform a manual initial alignment if necessary (using the "Transforms" module to roughly overlap volumes). Verify results by overlaying images with different colors. If misalignment is detected, consider adjusting Elastix parameters or using a different preset (e.g., use rigid before deformable).
	**b)** The registration module errors out.	**b)** Presence of scanning artifacts or irrelevant structures.	**b)** Crop or clean images before registration using relevant Slicer modules (e.g., Volumes Clip).
	**c)** Transforms not applied post-registration.	**c)** The user forgot to apply transforms to the moving image.	**c)** Explicitly apply the transform in the Data module post-registration.
5. CT Anatomical Segmentation	**a)** Poor segmentation results or significant distortion.	**a)** Inappropriate masking or threshold settings.	**a)** Disable unnecessary masking or thresholding.
	**b)** Hemorrhage segmentation includes bone or misses a lesion.	**b)** Using a simple threshold can be tricky: acute hemorrhage density may overlap with bone. If the threshold is set too high, it excludes parts of the hematoma; if it is set too low, it incorporates bone or other tissues.	**b)** Use a combined approach for hemorrhage: start with a threshold (around the typical blood HU) but then refine it. Employ the "Grow from Seeds" tool by seeding inside the hemorrhage and in adjacent brain tissue to separate it. After initial segmentation, remove obvious bone pieces by subtracting segments or using the scissors tool (limit region to intracranial area). Viewing in all planes helps ensure only the hemorrhage is captured.

Continued on next page

Table S3. – continued from previous page

Step	Problem	Possible Cause	Recommended Solution
	**c)** Incomplete bone segmentation.	**c)** The CT bone threshold might not pick up low-density bone or might exclude thinner bone sections. Conversely, artifacts (such as metal or noise) might create false positives.	**c)** Adjust the threshold to include the full range of cortical and trabecular bone (e.g., 200-1000 HU). Use the "Islands" tool with "Keep largest island" to remove tiny floating bits of noise. If the skull has holes (e.g., sinuses or surgical defects), that may be expected; to create a closed model, apply the "Surface Wrap Solidify" effect after segmentation to fill unintended gaps.
	**d)** Fiducial markers are unclear in CT.	**d)** Similar density structures obscured markers or were outside the scanned region.	**d)** Confirm scanning region includes markers; manually refine segmentation if necessary. Use a focused approach: isolate the region where markers are (e.g., outer scalp areas). Lower the threshold slightly to pick up marker material if it is low-density, or even segment it manually (paint or draw around the marker) if automatic methods fail. It can help to invert the window or level to spot markers.
6. Structural MRI Anatomical Segmentation	**a)** Misaligned lesion segmentation on MRI.	**a)** Applied transforms influencing image space.	**a)** Confirm transforms during the model export step; no need for immediate correction.
	**b)** Poor voxel set generation or severe distortion.	**b)** Non-orthogonal grid due to transforms; unintended masking or thresholding.	**b)** Confirm that no transformation has been applied to the source volume; turn off any inappropriate masking.
	**c)** Ventricular segmentation is irregular or leaks into the brain.	**c)** Similar intensity between CSF and adjacent structures.	**c)** Draw initial seeds carefully: one inside the ventricle CSF and one in the surrounding brain, across multiple slices. After using Grow from Seeds, if the results are rough, apply a slight smoothing: the "Smoothing" effect (Gaussian 1 mm) can improve contours. Remove any stray bits that leaked into the brain tissue using the Paint/Eraser in those areas.
	**d)** Venous segmentation misses small vessels.	**d)** Using a high threshold for venous sinuses captures the large sinuses but can miss smaller tributary veins; if the threshold is lowered too much, segmentation floods into surrounding tissue.	**d)** Segment in two passes: first, use a higher threshold to get the main venous sinuses. Then, for tributaries, use a lower threshold as suggested, but restrict the ROI to the vicinity of the sinus (to prevent leakage). You can do this by using the Scissors tool in rectangular mode to limit the area before thresholding. After thresholding small veins, immediately use "Islands" (keep largest) to eliminate noise. Combining these segments yields a more complete venous tree.
	**e)** Arterial segmentation includes non-vessels.	**e)** Bright structures like calcifications or fat (in the skull base) might be included when thresholding for arteries on MR, or the user may not set the ROI, leading to other areas being segmented.	**e)** Focus on one vessel territory at a time (the protocol divides ICA, ACA, MCA, VBA). Use ROI boxes or the "Mask volume" feature to isolate the region (e.g., around the Circle of Willis for ICA/MCA). After thresholding, remove obvious non-vessel bits: for example, if the internal jugular vein or bright dura got included, erase those using Scissors in Slice view. It's helpful to verify in 3D – true vessels will form continuous tubular shapes, whereas artifacts will look irregular.
	**f)** Failed skull stripping.	**f)** The Swiss Skull Stripper might not work if the template isn't loaded or if the MRI orientation is incompatible, or it completes but leaves residual skull.	**f)** Ensure the brain extraction template was downloaded and converted to a labelmap as instructed. If the skull stripping fails, check the console logs for errors (could be memory or a missing file). Try adjusting the initial alignment of the atlas to your data (the Slicer module may allow a rough transform). If slight scalp pieces remain, you can manually erase them or use a threshold just above CSF intensity to remove non-brain.
	**g)** The advanced vessel segmentation is poor.	**g)** The subtraction of pre- and post-contrast T1 images may not highlight vessels if the timing or contrast is suboptimal, or if the images aren't perfectly aligned, the subtraction will create artifacts.	**g)** Verify that the same anatomical region is being subtracted – the T1 and T1-CE must be well-registered (if not, apply the earlier transform or re-register them). If vessels still don't stand out, consider adjusting contrast: sometimes multiplying the subtracted volume by itself (squaring, as done) is sensitive. Ensure the threshold in this step is set appropriately (it might differ from the simpler threshold in step 6.4). If small arteries are still missed, you may need to manually add them or adjust the window/level to see if they are enhanced.
	**h)** Fiducial markers are invisible or not segmented.	**h)** MRI-visible fiducial markers (if used) can be very faint or only show on one sequence (e.g., maybe only on T1-CE or T2* as voids). The user might not see them to segment, or thresholding might not catch them.	**h)** Identify which MRI sequence shows the markers best – often T1-CE may show them as voids or bright spots if they contain contrast. If they are not visible, it may be that MRI markers were not present (sometimes, only CT markers are used). If they are present but faint, use manual segmentation: scroll to the slice where you expect the marker (based on CT positions) and use a small paint brush to label it. You can also try a targeted threshold just around the marker region (draw an ROI around where the marker should be, then threshold within that). After segmentation, check that the count matches the CT markers count.
7. White Matter Fiber Tractography	**a)** Tensor estimation fails, or no fibers are generated.	**a)** Missing diffusion metadata or incorrect seeding.	**a)** Confirm diffusion data is imported correctly; ensure no transform is applied to diffusion data.

Continued on next page

Table S3. – continued from previous page

Step	Problem	Possible Cause	Recommended Solution
	**b)** Software crashes due to memory limits.	**b)** An intensive computational task exceeds resources.	**b)** Allow enough time: whole-brain tractography can take 10–15 minutes, during which Slicer might appear unresponsive. Wait for completion or try a smaller region test to ensure it works. It's recommended to save the scene (or at least the diffusion data) before running tractography. Using the latest stable Slicer version and the UKF extension is also advised, as updates often improve performance and stability.
	**c)** The tractography result is difficult to visualize (too dense or unclear)	**c)** Whole-brain tracts create a dense "spaghetti" that's hard to interpret; default visualization might be thin lines blending.	**c)** Use the Tractography Display module to improve visualization: switch to "Tube" or thick line mode to see individual fibers. Apply coloring by bundle if possible (some tools color tracts by orientation). To focus, segment out specific pathways (as done in steps 7.2, 7.3): use ROI Markups to select only tracts passing through a region (e.g., cerebral peduncle for CST). This extraction (make a separate fiber bundle) will greatly clarify the view of, say, the corticospinal tract versus the rest. Hide the whole-brain tract when viewing specific tracts.
	**d)** Important fiber bundle not found (e.g., optic radiation appears incomplete)	**d)** Diffusion tractography might miss some tracts due to algorithm limitations or suboptimal seeding (optic radiations often require careful seeding near LGN and visual cortex).	**d)** Manually seeding: place inclusion ROIs in known start/endpoint regions (for optic radiation, around lateral geniculate nucleus and occipital lobe) and run tractography constrained to those (UKF allows seeding in ROI if configured). Alternatively, adjust algorithm parameters (e.g., allowing more curvature or using multiple fibers per voxel models for complexly curving fibers). Cross-check that the DWI quality is good; if motion artifact or low resolution is present, some tracts might not reconstruct well. Using a higher-order tractography method (if available) can sometimes recover missing fibers.
8. Surgical Trajectory Planning	**a)** AC–PC alignment seems incorrect (brain not properly reoriented)	**a)** The anterior/posterior commissure points might have been misidentified or placed off the true landmarks, resulting in a bad transform.	**a)** Re-check the AC and PC point placements: they should lie exactly at the center of the AC and PC in the midsagittal plane. Use the orthogonal slice views: in sagittal, pinpoint AC (at the fornix/lateral ventricle level) and PC (just above the superior colliculi). If necessary, adjust the points and reapply the ACPC transform. Verify by seeing that axial slices are roughly parallel to the AC–PC line after transform (brain should not look tilted). A slight error in selecting these points can lead to a noticeable tilt, so precision is crucial.
	**b)** Cranial landmark markers (Kocher's, Keen's, etc.) are placed inaccurately	**b)** These standard points have specific anatomical definitions (e.g., Kocher's point is a certain distance from midline and bregma). User error or patient-specific anatomy might cause misplacement.	**b)** Provide clear definitions in the protocol: e.g., "Kocher's point: 2–3 cm lateral to midline at the level of the coronal suture". In the planning step, after marking, double-check by measuring distances on the model (use the Ruler tool to ensure left-right symmetry and a reasonable distance from the midline or anatomical landmarks). If the patient's anatomy is unusual, rely on imaging (e.g., for Frazer's point, ensure it's at the appropriate ventricular target seen on axial MRI). Correct any deviating marks before proceeding, since trajectories depend on these points.
	**c)** Confusion among multiple trajectories.	**c)** With several plans (endoscopic, craniotomy, EVD, etc.), the scene can become cluttered with many lines and points, leading to confusion about which line corresponds to which plan.	**c)** Organize and label: use distinct colors for each trajectory line (e.g., red for endoscopic, blue for EVD, etc.) and name the markup nodes clearly (e.g., "Trajectory_EVD"). It may help to keep only one trajectory visible at a time while planning it (hide others). Document the plans: add a note in the protocol output describing which point set is entry vs target for each plan. This reduces mix-ups and ensures that during surgery, each plan is identifiable.
	**d)** The craniotomy plan misaligns with the intended trajectory.	**d)** If the planned bone flap (Step 8.5) is too small or mis-positioned relative to the trajectory, the surgical tool might not reach the target. This can occur if the user selects the incorrect center for the craniotomy.	**d)** Ensure the bone flap (circular or otherwise) is centered at the intersection of the trajectory with the skull. In 3D, visualize the trajectory line hitting the inner table of the skull – the craniotomy should be planned around that point with enough diameter for instrument maneuvering. If the hematoma is deep, consider a slightly larger diameter to allow angle adjustments. The protocol's use of the "Introducer" segment can help confirm that the bone window covers the introducer's path completely. If misaligned, reposition and possibly enlarge the planned flap segment, then regenerate the model.
	**e)** EVD trajectory misses the ventricle.	**e)** The EVD path (Step 8.6) might not enter the ventricle if the target or angle is off, especially in a distorted ventricle (due to mass effect).	**e)** After plotting the EVD line from Kocher's point, check the target in axial and coronal MRI – is it in the frontal horn of the lateral ventricle? If it's slightly off (too anterior or posterior), adjust the trajectory endpoint into the ventricle center (you can place a temporary markup inside the ventricle as a visual target). Also, ensure the depth is sufficient – the line should extend a bit past the ventricle wall into the cavity. If brain shift is a concern (since the patient has a hematoma), aim slightly toward the ventricular center. A trajectory that nicks the ventricle might fail in practice, so aim for a good centric placement.

Continued on next page

Table S3. – continued from previous page

Step	Problem	Possible Cause	Recommended Solution
9. 3D Model Generation and Export	**a)** The generated surface model is of low quality or has missing pieces	**a)** When converting segmentation to surface (e.g., CT or MRI Models), the resolution of the segmentation may be low or there may be holes due to threshold gaps.	**a)** Increase segmentation geometry resolution before model generation if needed (in the Slicer Segmentations module, you can oversample the master volume for finer detail). After initial model creation, inspect for holes: use "Closing" or the Surface Wrap Solidify effect to fill any gaps (especially for bone surfaces). For small missing pieces, you can also add them manually in segmentation and regenerate the model. Ensure each important structure (skull, lesion, vessels, etc.) was included in the segmentation that you exported – missing segments in the export will appear as missing parts of the model.
	**b)** Exported files are excessively large or slow.	**b)** High-resolution meshes (especially from whole-brain tractography or detailed vasculature) result in very large files that can lag or crash MR devices.	**b)** Simplify models before export: use the "Surface Toolbox" module to decimate meshes (reduce triangle count) while preserving shape. For example, a reduction to 50% or 25% of the original polygons can dramatically shrink the file size. Also, crop or split models if possible – e.g., you might not need the full skull at ultra-high detail for the MR demo; a decimated version will suffice visually. Test loading the models in a PC viewer (e.g., MeshLab or 3D Viewer) first to gauge performance. If necessary, export different layers as separate files to toggle them individually in the MR app, rather than one giant combined model.
	**c)** Misaligned models in MR view.	**c)** If CT and MRI models were exported separately, a transform might not have been applied equally, resulting in a registration mismatch in the MR scene (e.g., vessels not aligning with the skull).	**c)** Before export, harden all transforms on each segmentation or model so that they share a common coordinate space (e.g., patient anatomical space). A common mistake is exporting models from different coordinate systems – ensure all exports use the same reference (ideally after you've registered all images, everything is in the T1 space). If using an external tool or platform (such as Unity or the HoloLens app), confirm that it does not apply its scaling. Use a known dimension (like a ruler marker) to verify scale in MR. If misalignment is observed, re-export with corrected transforms or include a reference marker model that can be used to realign in the MR app. Additionally, be mindful of differences between left-handed and right-handed coordinate systems in subsequent software.
10. Fiducial Marker Localization	**a)** Combined marker cluster not separated into individual markers	**a)** The CT or MRI fiducial markers were segmented as one blob (e.g., if thresholding connected them via base material). If not split, you can't get individual marker positions.	**a)** Use the "Split islands to segments" tool immediately after segmenting markers. This will create separate segments for each physical marker. If the tool results in extraneous tiny segments (noise), delete those and keep the ones corresponding to real markers. Name each segment systematically (CT_Marker1, Marker2, etc.). If markers are too close and remain one island, you may need to manually draw a separating line in the segmentation to divide them.
	**b)** Different numbers of markers found in CT vs MRI	**b)** A marker might not be visible in MRI (e.g., it fell off before the MRI, or there is no signal due to an artifact), leading to 6 markers in CT but possibly only 5 in MRI.	**b)** If a marker is truly missing in MRI, you'll have an unequal set for registration – in this case, rely on surface matching (Step 11) more heavily, or remove that marker from the CT set when computing the transform (so both sets match). Double-check MRI sequences (sometimes markers show in one sequence but not another). If it's visible in some MRI (say T1-CE) but you segmented on T1WI, segment that particular one on the other sequence and transform it over. In summary, ensure the point count matches by either finding the missing marker or excluding the extra one; otherwise, pairing for registration will be wrong.
	**c)** Marker centroids are computed inaccurately.	**c)** If a segmented marker has an irregular shape or partial volume, the calculated centroid might not reflect the true physical center (especially if only part of the marker was segmented).	**c)** After segmentation, it can help to slightly dilate and then erode the marker segment to make it more solid (removing noise) before centroid calculation. Use Slicer's "Measurements" or "Labelmap Statistics" to get centroid coordinates. Alternatively, place a fiducial point manually at the center of the marker blob in the image (this might be more accurate if segmentation is rough). Compare the manual and automatic centroids to ensure they're reasonable. Any large discrepancy indicates the segment might be incomplete – refine the segment shape in that case. In any case, unreliable or suspected unreliable markers should be discarded.
11. Surface Registration Parameterization	**a)** The face surface mesh has holes or extra artifacts.	**a)** CT face extraction can accidentally include external objects (pillow, headrest) as part of the surface, and the MRI-based surface may have holes where there was no signal (e.g., around eyes or hair). These cause errors in parameterization.	**a)** Manually clean the surface segments: remove any non-human artifacts from the CT segmentation (you might see flat patches from a table or mask – erase those). For holes in the MRI surface, you can interpolate across the gap by drawing a patch in segmentation or using "Close holes" in the surface model. Additionally, decimate the meshes to a reasonable size and smooth them slightly so that ICP algorithms aren't thrown off by noise. A cleaner, simpler surface (just the general contour of the face or head) often yields a more robust registration.
12. Laser Projection Parameterization	**a)** Non-orthogonal or incorrectly positioned laser lines.	**a)** Reformat mode is causing incorrect slice positioning.	**a)** Set slices to standard axial, sagittal, and coronal views; activate slice intersections.
	**b)** Laser lines are visually confusing in 3D.	**b)** Lines are difficult to discern or are obstructed by other models.	**b)** Convert lines to clear models; limit length and distinguish lines by color for clarity.

Continued on next page

Table S3. – continued from previous page

Step	Problem	Possible Cause	Recommended Solution
13. Physical Validation Model Construction	**a)** The phantom model has unrealistic features.	**a)** The merged "physical twin" might have artifacts: for instance, combining skin, bone, markers, etc., could yield a model with jagged edges or internal structures poking through the surface.	**a)** Use the "Surface Wrap Solidify" tool to create a unified outer surface. For example, you might combine the segmented skin and markers, then wrap them to create a single, closed surface that has markers bulging where they should. If internal parts (like skull or ventricles) are included but shouldn't affect the external shape, exclude them from the wrap that defines the outer surface. The idea is to simulate a realistic surface of the head + markers. Any non-realistic protrusions (due to segmentation noise) should be trimmed or smoothed before finalizing the phantom model.
	**b)** Difficulty generating the model due to complexity.	**b)** Computational intensity.	**b)** Reduce model complexity by turning off unnecessary 3D visualization during generation.
14. Virtual Reference Model Generation	**a)** Quadrant labeling confusion.	**a)** Unclear labeling of quadrant divisions.	**a)** Regularly use the Models module for verification; correlate quadrant labeling with surgical planning.
15. Navigational Accuracy and Performance Analysis	**a)** Fiducial pair error calculation (FLE/TRE) seems wrong	**a)** Suppose the corresponding fiducial points between CT and MRI were mismatched or labeled incorrectly. In that case, the computed Fiducial Localization Error (FLE) or Target Registration Error (TRE) will be invalid (possibly very large or nonsensical).	**a)** Double-check the pairing of fiducials: the "ordered fiducial pairs" in step 15.1 must truly correspond one-to-one (e.g., CT marker A with MRI marker A). Use consistent naming or an ordering scheme when extracting centroids (Step 10) so that you combine them correctly. In Slicer's Fiducials-to-Model Distance or other error calculation module, ensure you input the correct point sets. If an error looks way off (e.g., one marker shows a 50 mm error while others are 2 mm), likely a pairing is wrong or that marker wasn't visible in one modality (thus a wrong point was used) – fix the pairing and recompute.
	**b)** Computed error metrics (values) are higher than expected	**b)** Even if pairing is correct, you might see larger TRE/FLE than anticipated (e.g., > 10 mm) when you expected approx. 1–2 mm. Causes can include a systemic bias in registration (all points slightly off in one direction) or one quadrant of the head having worse alignment.	**b)** Investigate pattern: if all fiducials have a consistent offset, the registration transform might have a slight rotation/scale error – using surface alignment (Step 11) plus markers could introduce that. One solution is to perform a fine registration just on the markers: use a landmark registration tool to tweak the transform. If one region (e.g., the posterior head) has higher errors, it suggests that deformation is not accounted for (MRI and CT may have slight patient movement differences). Note this limitation in results – it may not be fully correctable. For analysis, you may remove an outlier point and recalculate the average TRE (report both the original and adjusted values).
	**c)** Extrapolated error field (15.3) is noisy or unclear	**c)** The creation of an "extrapolative error field" involves generating a dense grid of points and measuring error – if those points (P-set) aren't well-distributed or if the transform is non-linear, the visualization might look random or too dense to interpret.	**c)** Ensure the P-Set generation step (cloning centroids and extending to a grid) is done correctly. You might want to limit the region for analysis – e.g., within the convex hull of your markers, since extrapolation far outside can be unreliable. Once you have the error vectors, use a suitable scale and visualization (such as arrows or a color map on a model). If it's too noisy, consider smoothing the field or computing a regional average error. The reliability analysis should highlight broad trends (such as errors increasing toward one side); if noise obscures this, reduce the point density or bin the data into areas (perhaps those scalp quadrants) and compute the mean errors per quadrant for clarity.
	**d)** Global error visualization (15.4) arrows are too small/large or mis-scaled	**d)** In the Transforms module's display of error vectors, it's possible the arrows representing error are not visible or all overlapping if the scale is off (e.g., a 2 mm error arrow might be tiny in a scene scaled in mm).	**d)** Adjust the visualization scale: In the Transforms Display, there is usually a glyph size or scale factor – increase it until arrows are visible. Conversely, if the arrows are too large and clutter the view, scale them down. Also, color-code the arrows by magnitude (if the tool allows) or note the length differences. Ensure you've switched the transform direction appropriately: you want to visualize the error from one modality to the other. If needed, manually create a vector model by subtracting coordinates to double-check that the displayed arrows are correct.
	**e)** Local error at structures (15.5/15.6) yields zero or a strange result	**e)** For structure-specific accuracy, the protocol includes steps such as duplicating a lesion and applying a transform, then comparing the overlap or distance. If the result is zero error or very high, something might be wrong in the process.	**e)** Make sure you applied the exact displacement field transform to the duplicated structure. If you accidentally compared the same lesion to itself, you'd get zero error (false indication). On the other hand, if you applied a wrong transform (or none), you're comparing unrelated objects, giving meaningless high values. Use quantitative measures, such as calculating the Dice coefficient for overlap or measuring surface distance. A very low Dice score or a very high distance indicates the misapplication of the transform. Redo the step carefully with the correct moving data.
	**f)** Intrinsic parameter computation (Step 15.7) is confusing or not provided	**f)** Insufficient guidance on computing this metric	**f)** If this step is optional or for analysis reporting only, mention that skipping it doesn't affect the workflow; it's for the documentation of accuracy.
