# Supplementary material for: Comprehensive protocol for mixed reality visualization and navigation using 3D Slicer
Source: PLoS One. 2026 Mar 31;21(3):e0343997. doi: 10.1371/journal.pone.0343997 (PMC13038006; doi:10.1371/journal.pone.0343997)
Supplement: S4 Appendix — Provided are detailed mathematical definitions of the accuracy metrics, displacement field computation, and additional visualization examples. (PDF) [file pone.0343997.s004.pdf]

S4 Appendix. Technical details of the accuracy assessment framework

S4.1 Parameterization of fiducial sets and accuracy domains

Step 15 provides a quantitative assessment of the MRN system's spatial accuracy using the twin models as ground truth references. After registering the MRN system to the static twins, the system's reported spatial coordinates are compared with the known reference positions. A predefined set of fiducial markers (the "Centroid Set", C-Set) serves as the ground truth for evaluating errors in both physical space (actual patient/phantom coordinates, P-Set) and virtual space (the corresponding points in the virtual model, V-Set). Errors are assessed at the reference fiducial locations (interpolation errors) as well as across the broader workspace (extrapolation errors). The interpolation error at each fiducial is quantified directly (e.g., using distance-measurement tools such as "*Q3DC*" or "*Fiducial-to-Model Distance*" module). To assess the extrapolation error, a fiducial-based registration is performed between the ground truth markers and the navigation system's indicated positions (using the "*Fiducial Registration Wizard*" [1]), and the resulting displacements are analyzed (see Fig 1) for the definition of these error domains). Together, these metrics characterize the overall spatial precision of the navigational registration procedure and constitute the system's accuracy performance.

S4.2 Definition and interpretation of accuracy metrics

Step 15 produces quantitative and qualitative accuracy assessments. Spatial registration errors are quantified by comparing coordinates of physically acquired points (P-Set) and virtually perceived points (V-Set) against ground-truth centroid references (C-Set) (see Fig 2A). The quantitative framework involves two main domains of error metrics: virtual-to-physical ($C \to P$) and virtual-to-perceptual ($C \to V$) comparisons (see detailed definitions in Table 1). Virtual-to-physical metrics assess spatial discrepancies between virtual holographic targets and their corresponding physical points, directly reflecting clinical accuracy. Virtual-to-perceptual metrics assess discrepancies arising solely within the visualization system, highlighting perceptual biases introduced by holographic rendering and display limitations. All metrics can be divided into two categories based on their calculation methods: interpolation error, which represents local accuracy at directly measured landmarks; and extrapolation error, which reflects global accuracy throughout the navigational space.

To enable future studies to perform quantitative performance comparisons using this evaluation framework, the present protocol incorporates quantitative results from our group's previously published companion studies. It provides them here as standardized benchmarks [2]. It is important to distinguish between the "accuracy assessment" itself and the "accuracy performance" of any specific system or registration algorithm. The protocol defines a generalizable evaluation scheme with related metrics in Table 1, applicable to any MRN registration paradigm. The quantitative results reported in the

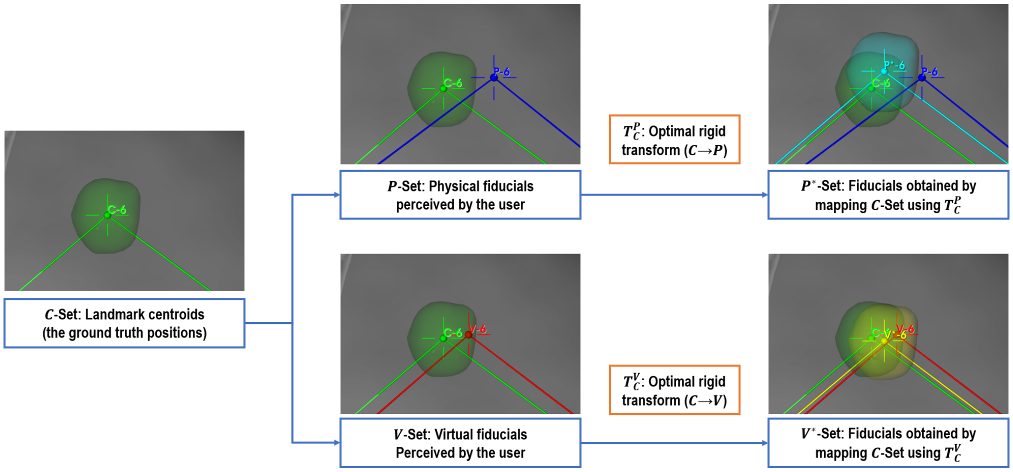

Fig 1. Parameterization of fiducial sets and the rigid transformation-based MRN accuracy assessment method. Based on segmented fiducial markers (translucent green), the accuracy assessment framework defines landmark centroids (C-Set, green) as the ground truth, physical fiducials (P-Set, dark blue), and virtual fiducials (V-Set, red), both perceived by the user. The framework assesses accuracy across two distinct spatial mapping domains: the physical registration domain ($C \to P$), which measures discrepancies between ground truth fiducials and user-perceived physical positions; and the virtual perception domain ($C \to V$), which measures discrepancies between ground truth fiducials and user-perceived virtual positions. Optimal rigid transformations (T_C^P and T_C^V) map the C-Set onto the P-Set and the V-Set, respectively, generating transformed fiducial sets (P^*-Set and V^*-Set, cyan and yellow) and markers (translucent cyan and yellow) for comparative accuracy evaluation.

main text therefore serve as reference benchmarks for assessing reproducibility and internal reliability, rather than representing fixed performance limits.

S4.3 Deviation and displacement field evaluation and visualization

Additionally, comprehensive visualizations effectively illustrate local and global accuracy. Displacement fields and anatomical deviations are visualized through intuitive color mappings in both 2D and 3D views, highlighting spatial discrepancies (see Fig 2B– 2D). The quantitative assessment framework also computes structural metrics, such as centroid locations, volumes, depths, and overlap indices, for specific anatomical regions, providing a robust quantitative basis for localized accuracy evaluation, as visualized in Fig 2E and 2F. Collectively, these visualizations and quantitative data provide actionable feedback to optimize, validate, and implement the MRN system effectively in clinical settings.

S4.4 Empirical patterns and internal consistency checks

Beyond serving as reference benchmarks, the quantitative results also reveal several characteristic empirical patterns inherent to the defined parameter framework, which can assist users in evaluating the internal consistency of their own systems:

Table 1. Definition and interpretation of error metrics for quality assessment

Metric†	Domain	Definition	Type‡	Computation	Interpretation
FLE	$C \to P$	User perception of localization of fiducials in physical space	Intp.	Approx. via tre	User uncertainty in locating physical fiducials
TRE	$C \to P$	Virtual- to physical displacement at targets after the registration	Intp.	$P - C$	Overall system accuracy of virtual-to-physical registration
T_C^P	$C \to P$	The optimal rigid transform from ground truth fiducials to user-perceived physical fiducials	Extp.	Least-squares fitting $C \to P$	Virtual-to-physical displacement field
FN	$C \to P$	Measure of virtual-to-physical displacement field magnitude	Extp.	$\|T_C^P - I\|_F$	Overall magnitude of displacement field
FRE	$C \to P$	Difference between the user-perceived physical fiducials and their registered positions after the optimal transformation.	Extp.	$P - P^*$, i.e., $P - T_C^P \cdot C$	Reliability of the extrapolated displacement field
R, R_x, R_y, R_z	$C \to P$	Rotation component (Euler angle) of the optimal transformation	Extp.	The Euler angle of T_C^P	Orientation difference between virtual and physical spaces after registration
t, t_x, t_y, t_z	$C \to P$	Translation component of the optimal transformation	Extp.	The last column of T_C^P	Positional offset between virtual and physical coordinate origins
DSC	$C \to P$	Dice Similarity Coefficient, measuring volumetric overlap	Extp.	$\frac{2\|X \bigcap Y\|}{\|X\|+\|Y\|}$	Quantitative measure of segmentation overlap accuracy.
HD_{95}	$C \to P$	95th percentile Hausdorff Distance	Extp.	$\overset{95}{\max_{x \in X}} \left(\min_{y \in Y} \|y - x\|_2 \right)$	Maximum boundary discrepancy at the 95th percentile level between virtual and physical structures
fle	$C \to V$	Difference between user-perceived virtual fiducial location and rendered virtual fiducial location (not directly measurable)	Intp.	Depends on the optical parallax effects of HMD	Uncertainty from HMD tracking or holographic drift, i.e., the ambiguity in rendering and tracking alignment
tre	$C \to V$	Displacement of user-perceived virtual fiducials to their ground truth	Intp.	$V - C$	Overall accuracy of user's virtual-space 3D perception
T_C^V	$C \to V$	The optimal rigid transform from ground truth fiducials to user-perceived virtual fiducial set	Extp.	Least-squares fitting $C \to V$	User's perceptual displacement field
fn	$C \to V$	Measure of user's perceptual displacement field magnitude	Extp.	$\|T_C^V - I\|_F$	Overall magnitude of user's perceptual displacement field
fre	$C \to V$	Difference between the user-perceived virtual fiducials and their registered positions after the optimal transformation.	Extp.	$V - V^*$, i.e., $V - T_C^V \cdot V$	Consistency and repeatability of user's virtual interactions

† $FLE \mid fle$ = fiducial localization error; $FN \mid fn$ = Frobenius Norm; $FRE \mid fre$ = fiducial registration error; $TRE \mid tre$ = target registration error. ‡Intp. = Interpolation; Extp. = Extrapolation.

1. Virtual–physical metrics (e.g., FRE, FN) are typically greater than their virtual–to-perceptual counterparts (fre, fn), because the registration process in

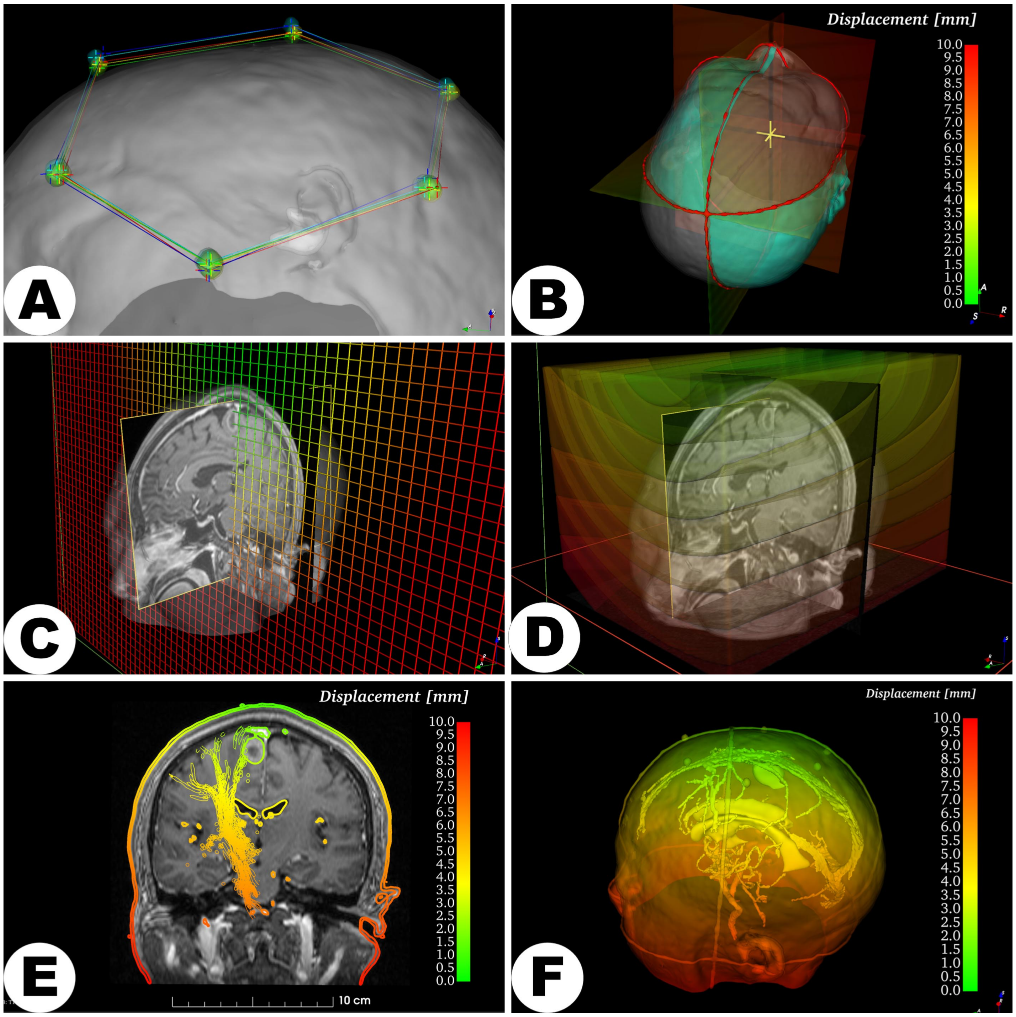

Fig 2. Representative examples of deviation and displacement field evaluation and visualization. A: Comparison and visualization of ordered fiducial sets. B: Error evaluation using scalp quadrants (SQs), laser projection lines (LPLs), and reference planes. C: Visualization of the displacement field for the special 2D slicer. D: 3D visualization and parameterized interpolation of the displacement field. E: 2D visualization of displacement for specific anatomical structures. F: 3D visualization of displacement for specific anatomical structures.

the physical domain introduces additional compound errors originating from registration itself, rather than from purely geometric or perceptual factors.

2. The TRE generally exceeds both FLE and FRE. In this protocol, TRE represents a user-perceived interpolated error. Since the final MxR visualization is projected onto the user's retina, the ability to perceive even subtle misalignments between physical and virtual structures indicates a higher-level integrated perceptual accuracy encompassing both measurement and visualization precision. In contrast, FLE and FRE capture more localized measurement deviations within single operational steps, reflecting limited sources of error and thus yielding smaller numerical values.

3. The translation magnitude t should not exceed the FN. When rotational

misalignment R is small (typically less than 5°), t approximates FN, which is consistent with the mathematical definition of the transformation matrix norm.

Recognizing these empirical patterns provides practical guidance for evaluating whether new MRN implementations yield results consistent with expected system behavior and measurement reliability. Future users may apply this framework to other registration strategies or imaging configurations to generate comparable quantitative accuracy metrics. By reporting results using unified definitions and error domains, subsequent studies will be able to quantitatively benchmark their performance against these standardized references, thereby promoting methodological consistency and reproducibility in MRN system validation.

References

1. Ungi T, Lasso A, Fichtinger G. Open-source platforms for navigated image-guided interventions. Medical image analysis. 2016;33:181–186.

2. Qi Z, Jin H, Wang Q, Gan Z, Xiong R, Zhang S, et al. The feasibility and accuracy of holographic navigation with laser crosshair simulator registration on a mixed-reality display. Sensors. 2024;24(3):896. doi:10.3390/s24030896.
