# Supplementary material for: Comprehensive protocol for mixed reality visualization and navigation using 3D Slicer
Source: PLoS One. 2026 Mar 31;21(3):e0343997. doi: 10.1371/journal.pone.0343997 (PMC13038006; doi:10.1371/journal.pone.0343997)
Supplement: Attachment — Submitted filename: Rebuttal.pdf [file pone.0343997.s005.pdf]

Reply to the Academic Editor and the Reviewer

Manuscript ID: PONE-D-25-39628
"Comprehensive protocol for mixed reality visualization and navigation using 3D Slicer"
Ziyu Qi, Christopher Nimsky, and Miriam H. A. Bopp
PLOS ONE

Editor, comment #1

Please ensure that your manuscript meets PLOS ONE's style requirements, including those for file naming. The PLOS ONE style templates can be found at (websites)

Our response #0.1

We appreciate the editor's reminder and have checked to ensure that the manuscript complies with PLOS ONE's formatting and file naming guidelines.

Change to Text: N/A

Editor, comment #2

In your Methods section, please provide additional information regarding the permits you obtained for the work. Please ensure you have included the full name of the authority that approved the field site access and, if no permits were required, a brief statement explaining why.

Our response #0.2

We thank the editor for the reminder. This study did not involve any fieldwork, patient recruitment, or access to clinical sites. All analyses were performed on a publicly available, fully anonymized dataset [Qi Z et al. (2024), DOI: https://doi.org/10.6084/m9.figshare.24550732.v6] released under a CC BY 4.0 license, and on 3D-printed phantoms derived from these data in a laboratory setting. Therefore, no field site access permissions or collection permits were required. We have added an explicit statement in the Methods section to clarify this.

Change to Text: **[Page 5, line 130]** Because the study involved no fieldwork, patient recruitment, or access to clinical or experimental sites, no field site access permissions or collection permits were required.

Editor, comment #3

We noted in your submission details that a portion of your manuscript may have been presented or published elsewhere. "Table 1 and Figure 8 are reproduced under CC-BY 4.0 license, credit:

Table 1: Amla et al. (2024), published in Journal of Digital Imaging (37(1):386–401, DOI: 10.1007/s10278-023-00912-y)

Figure 8: Qi Z et al. (2024) Head model dataset for mixed reality navigation in neurosurgical interventions for intracranial lesions. Sci Data 11: 538. doi: https://doi.org/10.1038/s41597-024-03385-y ". Please clarify whether this [conference proceeding or publication] was peer-reviewed and formally published. If this work was previously peer-reviewed and published, in the cover letter please provide the reason that this work does not constitute dual publication and should be included in the current manuscript.

Our response #0.3

We thank the editor for the attention to this matter. **Table 1** was reproduced from *Amla et al.*, *Journal of Digital Imaging* (2024, peer-reviewed) under the Creative Commons Attribution 4.0 International (CC BY 4.0) license. The table summarizes publicly available comparative data on software performance and was reused without modification solely to provide contextual background on the selection of open-source software. The objectives of that review and the present study are entirely different. **Figure 8** was reproduced from *Qi et al.*, *Scientific Data* (2024, peer-reviewed) under the CC BY 4.0 license. It is included only to

illustrate the concept of *physical and virtual twins* (“reference object principle”) described in the current Methods section. The *Scientific Data* paper focuses on constructing a public MRN dataset for research use. In contrast, the corresponding section of this protocol explains the generation of physical and virtual twins (*Steps 13–14*) and their use for accuracy assessment (*Step 15*). Both materials are fully credited, unmodified, and reused in accordance with open-access CC BY 4.0 terms. They serve purely illustrative and methodological reference purposes and do not constitute new or overlapping results. This manuscript presents an original, comprehensive laboratory protocol that has not been published elsewhere. Therefore, inclusion of these open-licensed materials does not constitute dual publication.

Change to Text: N/A

Editor, comment #4

We note you have not yet provided a protocols.io PDF version of your protocol and/or a protocols.io DOI. When you submit your revision, please provide a PDF version of your protocol as generated by protocols.io (the file will have the protocols.io logo in the upper right corner of the first page) as a Supporting Information file. The filename should be S1_file.pdf, and you should enter “S1 File” into the Description field. Any additional protocols should be numbered S2, S3, and so on. Please also follow the instructions for Supporting Information captions [https://journals.plos.org/plosone/s/supporting-information#loc-captions]. The title in the caption should read: “Step-by-step protocol, also available on protocols.io.”

Please assign your protocol a protocols.io DOI, if you have not already done so, and include the following line in the Materials and Methods section of your manuscript: “The protocol described in this peer-reviewed article is published on protocols.io (https://dx.doi.org/10.17504/protocols.io.[...]) and is included for printing purposes as S1 File.” You should also supply the DOI in the Protocols.io DOI field of the submission form when you submit your revision.

Our response #0.4

We thank the editor for this reminder. The complete step-by-step protocol has now been formally published on protocols.io and assigned the DOI [https://dx.doi.org/10.17504/protocols.io.kqdg327p1v25/v1]. The official PDF version (with the protocols.io logo) has been uploaded as S1 File according to journal guidelines. In the “Materials and Methods” section, we have replaced the previous private link with the required citation.

Change to Text: **[Page 7, line 243]** The protocol described in this peer-reviewed article is published on protocols.io (https://dx.doi.org/10.17504/protocols.io.kqdg327p1v25/v1) and is included for printing purposes as S1 File.

Change to Text: **[Page 20, line 645] S1 File**: A step-by-step protocol, also available on protocols.io (https://dx.doi.org/10.17504/protocols.io.kqdg327p1v25/v1). (PDF)

Editor, comment #5

If the reviewer comments include a recommendation to cite specific previously published works, please review and evaluate these publications to determine whether they are relevant and should be cited. There is no requirement to cite these works unless the editor has indicated otherwise.

Our response #0.5

We carefully reviewed all reviewer-suggested references. Only works directly relevant to the topic, methodology, or context of this protocol were cited.

Change to Text: N/A

Reviewer #1, comment #1

Scope and Innovation within a Lab Protocol

Even allowing for the procedural focus of a Lab Protocol, the manuscript remains largely an integration of existing 3D Slicer modules (segmentation, surface modeling, fiducial-based registration). The unique methodological advance—beyond assembling known tools—is not sufficiently articulated.

Please clearly state what is genuinely novel (eg, a distinctive integration strategy, the static digital-twin validation framework, or standardized accuracy metrics) and explain why this consolidation itself represents a meaningful advance over existing Slicer tutorials or comparable platforms such as MITK or MeVisLab.

Our response #1.1

We thank the reviewer for this insightful comment. We agree that the methodological novelty of the protocol needed clearer articulation beyond the integration of existing 3D Slicer modules. In revision, we added a new subsection, "Core methodological contributions," and expanded "Comparison with related tools and resources" and "Positioning within the literature." These revisions clarify three key innovations: (i) standardized modular integration of imaging, segmentation, registration, and validation into a single reproducible workflow; (ii) a static digital-twin validation framework for non-contact quantitative accuracy testing; and (iii) a structured accuracy taxonomy for standardized accuracy reporting. In addition, Table 6 was added to illustrate the hierarchical three-layer architecture supporting the protocol's clarity, reproducibility, and adaptability.

Change to Text: [Page 18, line 567] Core methodological contributions

Most prior work in this field has focused on isolated components of the workflow, without providing an end-to-end, reproducible standard for MRN development and validation. In contrast, the present protocol introduces three key methodological advances that, collectively, establish a unified foundation for MxR–based neurosurgical research and training. First, it provides standardized, modular integration of data import, segmentation, registration, and validation steps into a single, reproducible workflow that can be executed entirely within 3D Slicer. Second, it establishes a static digital twin validation framework that enables non-contact, repeatable, and quantitative testing using publicly available datasets. Third, it defines a structured accuracy taxonomy—distinguishing interpolation and extrapolation domains across virtual–to-physical and virtual–to-perceptual mappings—that standardizes accuracy reporting and facilitates objective comparison among MRN systems (see Table 3). Together, these innovations transform previously fragmented procedures into a coherent, validated methodological standard that enhances reproducibility, lowers technical barriers, and supports interdisciplinary collaboration.

Change to Text: [Page 16, line 495] Beyond the integration of software extensions, the step-by-step protocol introduces a clearly defined three-layer hierarchical structure that enhances procedural clarity, reproducibility, and modular adaptability (see Table 6 and S1 File). At the core content layer, each section, step, and sub-step is explicitly numbered. It corresponds to specific video segments in the supplementary demonstration (see S2 Appendix) and provides detailed action lists, required materials, and expected outcomes for each procedure. The supportive informational layer complements these steps with contextual annotations—such as notes, safety information, and expected results—that ensure interpretability, safety, and standardized verification. At the organizational layer, the protocol defines dependencies among both steps and extension modules, enabling their flexible recombination into six specialized pipelines optimized for different application scenarios (Fig 3). Together, this hierarchical design (summarized in Table 6) ensures transparency, facilitates learning, and enables flexible reuse and extension across diverse clinical and research contexts.

Change to Text: [**Page 17, Table 6. Structural hierarchy of the step-by-step protocol based on the three-layer architecture.**]

Change to Text: [Page 16, line 517] For visualization and educational applications, most reported workflows follow a similar data-processing chain that includes sequence alignment, segmentation, 3D surface reconstruction, and model optimization to enhance realism. These processes commonly involve transforming multimodal medical image coordinates into an anatomically aligned space, segmenting relevant structures, converting label maps into geometric surface models, and applying smoothing or texturing to improve visual quality [59]. Some studies further enrich the models with advanced attributes to simulate tissue deformation [59], or introduce parametric nodes to quantitatively assess trainees' performance during simulated procedures [58]. Despite these technical advances, the underlying procedures are far from straightforward: they require close collaboration between experts in neurosurgery, anatomy, radiology, computer graphics, and engineering [59]. The resulting fragmentation of expertise and tools substantially limits efficiency and reproducibility [59]. Detailed step-by-step procedures are rarely disclosed, processing times and complexity are seldom reported, and open-source reproducibility is often lacking. The present protocol addresses these shortcomings through a modular, semi-automated design. It provides standardized parameters and

explicit operational guidelines that allow for human supervision throughout while maintaining efficiency and consistency. This combination of automation and user control enhances reproducibility, reduces the burden of interdisciplinary coordination, and lowers the technical barrier for both clinicians and developers.

Reviewer #1, comment #2

Quantitative Validation and Benchmarking

The protocol commendably includes a built-in accuracy-assessment framework (TRE, FLE, displacement fields, Dice coefficient, HD95) and describes static digital-twin validation in detail. Despite these definitions, the manuscript provides no aggregated multi-case accuracy statistics, inter-observer reproducibility data, or comparative benchmarking against commercial navigation systems or other open-source pipelines. The authors need to add at least limited multi-case results or explicitly reference companion studies that report quantitative performance using this protocol to substantiate reliability and reproducibility.

Our response #1.2

We thank the reviewer for this valuable comment. In response, we have expanded the section *"Use-case: MRN development, validation, and accuracy assessment"* to include quantitative benchmarking data (**Tables 4** and **Table 5**), clarify the distinction between the accuracy-assessment framework and algorithm-specific performance, and summarize empirical patterns derived from multi-case results.

Change to Text: **[Page 12, line 413]** To enable future studies to perform quantitative performance comparisons using this evaluation framework, the present protocol incorporates quantitative results from our group's previously published companion studies. It provides them here as standardized benchmarks [25]. It is important to distinguish between the "accuracy assessment" itself and the "accuracy performance" of any specific system or registration algorithm. The protocol defines a generalizable evaluation scheme with related metrics in Table 3, applicable to any MRN registration paradigm. The quantitative results reported below therefore serve as reference benchmarks for assessing reproducibility and internal reliability, rather than representing fixed performance limits.

In the referenced companion studies, a novel registration method based on a laser crosshair simulator (LCS) was developed and validated using 19 patient-specific head phantoms [25]. Aggregated accuracy results across all validation cases are summarized in Table 4, and detailed case-wise data are listed in Table 5. These values constitute the first systematically validated reference benchmarks for MRN accuracy established using this protocol. Because fle cannot be directly measured or computed, and because the transformation components (T_C^P, T_C^V, and their rotational and translation subcomponents, i.e., $R_{x,y,z}$ and $t_{x,y,z}$), depend on the imaging coordinate system and are thus not directly comparable across cases, these parameters are not reported here.

Beyond serving as reference benchmarks, these results also reveal several characteristic empirical patterns inherent to the defined parameter framework, which can assist users in evaluating the internal consistency of their own systems:

1. Virtual–physical metrics (e.g., FRE, FN) are typically greater than their virtual–to-perceptual counterparts (fre, fn), because the registration process in the physical domain introduces additional compound errors originating from registration itself, rather than from purely geometric or perceptual factors.

2. The TRE generally exceeds both FLE and FRE. In this protocol, TRE represents a user-perceived interpolated error. Since the final MxR visualization is projected onto the user's retina, the ability to perceive even subtle misalignments between physical and virtual structures indicates a higher-level integrated perceptual accuracy encompassing both measurement and visualization precision. In contrast, FLE and FRE capture more localized measurement deviations within single operational steps, reflecting limited sources of error and thus yielding smaller numerical values.

3. The translation magnitude t should not exceed the FN. When rotational misalignment R is small (typically less than 5°), t approximates FN, which is consistent with the mathematical definition of the transformation matrix norm.

Recognizing these empirical patterns provides practical guidance for evaluating whether new MRN implementations yield results consistent with expected system behavior and measurement reliability. Future users may apply this framework to other registration strategies or imaging configurations to generate comparable quantitative accuracy metrics. By reporting results using unified definitions and error domains, subsequent studies will be able to quantitatively benchmark their performance against these standardized references, thereby promoting methodological consistency and reproducibility in MRN system validation.

Change to Text: [**Page 14, Table 4. Quantitative benchmarking summary across all validation cases (n = 19).**]

Change to Text: [**Page 15, Table 5. Detailed case-wise quantitative benchmarking results.**]

Reviewer #1, comment #3

Static Digital Twin Limitation

The protocol relies exclusively on static digital twins for accuracy testing. Static models cannot capture intraoperative brain shift or dynamic anatomical changes—key challenges for neurosurgical navigation. Discuss the clinical implications of this limitation and outline potential extensions (eg, real-time sensor integration, dynamic or "shadow" digital twins).

Our response #1.3

We thank the reviewer for this insightful comment and agree that the lack of dynamic adaptability can limit the protocol's clinical relevance, as intraoperative brain shift and tissue deformation may compromise navigational accuracy and surgical safety margins. To address this, we have expanded the discussion in the "Limitations and future directions" section. The revised paragraph now analyzes the clinical implications of static digital twins and outlines potential solutions. Furthermore, we have incorporated an example of recent AI-based monocular reconstruction research, demonstrating that reliable 3D surface reconstruction from a single 2D intraoperative image can enable compact, inexpensive, real-time surface modeling. When combined with lightweight head-mounted displays, these approaches could support dynamic digital twins capable of tracking intraoperative deformation and assisting real-time surgical risk prediction.

Change to Text: [**Page 19, line 593**] Secondly, the current version is predominantly limited to static digital twins, representing fixed digital replicas without the capability for dynamic real-time synchronization (i.e., shadow twins) [66]. While static twins enable reproducible accuracy testing, they cannot account for intraoperative brain shift or tissue deformation, which can lead to progressive misalignment between holographic overlays and the actual surgical field. Such discrepancies can reduce navigational accuracy and affect surgical safety margins during resection. To address this limitation, future developments should integrate real-time sensor data, advanced finite element modeling, and AI-driven predictive algorithms. Recent research has demonstrated that AI-driven monocular reconstruction techniques enable reliable 3D surface reconstruction from a single 2D intraoperative image [59]. In these approaches, a deep-learning network predicts per-pixel depth maps from monocular photographs, which are subsequently converted into dense 3D point clouds [67], offering a compact and inexpensive route toward dynamic surface modeling. When combined with HMDs or lightweight cameras capable of continuous image capture, these approaches could generate dynamic digital twins that update in near real time [66], enabling the tracking of intraoperative tissue deformation and real-time surgical risk prediction, though at the expense of higher computational and implementation complexity.

Reviewer #1, comment #4

Positioning Within the Literature

Similar end-to-end MR workflows using 3D Slicer or related platforms have been described. The manuscript does not adequately compare its efficiency, accuracy, or ease of adoption with these prior efforts. Provide a critical, evidence-based comparison to related protocols like those I recommended below, highlighting concrete advantages such as reduced processing time, improved accuracy, or lower hardware costs. (12 References)

Our response #1.4

We sincerely thank the reviewer for this insightful comment. In response, we have added a new subsection entitled "Positioning within the Literature". This subsection critically analyzes and compares our protocol with the reviewer-recommended references and other published workflows on neurosurgical 3D visualization and navigation. The new text explicitly situates the present protocol within a comprehensive methodological framework that covers all three major application domains (i.e., preoperative planning, educational visualization, and intraoperative navigation) and highlights its specific advantages: 1) Detailed and openly documented workflow: provides clinicians and developers with a transparent, step-by-step process to manage technically complex procedures, thereby lowering the technical barrier to adoption; 2) Efficient use of publicly available data: reduces both development time and resource costs for research and system

prototyping; 3) Standardized and reproducible evaluation metrics: simplifies system performance assessment, enhances repeatability, and enables structured, comparable quantitative outputs across different MRN systems.

Change to Text: [Page 16, line 509] Positioning within the literature

Within the broader context of neurosurgical 3D visualization and navigation, existing studies can be broadly categorized into three domains: preoperative surgical planning [55-58], educational or rehearsal-based visualization [59,60], and intraoperative navigation systems [61,62], as well as hybrid approaches combining these purposes [63,64]. The present protocol is designed to be compatible with all of these applications, providing an end-to-end methodological framework that can be directly applied to surgical planning, training, and navigation validation.

For visualization and educational applications, most reported workflows follow a similar data-processing chain that includes sequence alignment, segmentation, 3D surface reconstruction, and model optimization to enhance realism. These processes commonly involve transforming multimodal medical image coordinates into an anatomically aligned space, segmenting relevant structures, converting label maps into geometric surface models, and applying smoothing or texturing to improve visual quality [59]. Some studies further enrich the models with advanced attributes to simulate tissue deformation [59], or introduce parametric nodes to quantitatively assess trainees' performance during simulated procedures [58]. Despite these technical advances, the underlying procedures are far from straightforward: they require close collaboration between experts in neurosurgery, anatomy, radiology, computer graphics, and engineering [59]. The resulting fragmentation of expertise and tools substantially limits efficiency and reproducibility [59]. Detailed step-by-step procedures are rarely disclosed, processing times and complexity are seldom reported, and open-source reproducibility is often lacking. The present protocol addresses these shortcomings through a modular, semi-automated design. It provides standardized parameters and explicit operational guidelines that allow for human supervision throughout while maintaining efficiency and consistency. This combination of automation and user control enhances reproducibility, reduces the burden of interdisciplinary coordination, and lowers the technical barrier for both clinicians and developers.

In navigation-oriented studies, most validation frameworks rely on paired virtual–physical models, or digital twins. The virtual model represents the patient's anatomy displayed to the user, whereas the physical counterpart can consist of cadaveric heads [59], patient-specific 3D-printed models [59], or the patient's own anatomy [61,62]. While these approaches are conceptually powerful, they face several practical limitations. Recruiting sufficient clinical cases for validation is time-consuming and requires extensive ethical approvals [39,65]. Commercially available head phantoms are costly, whereas low-cost plastic models often lack medical relevance [39,65]. Moreover, measurements using commercial navigation hardware are inherently invasive—placing physical probes, fiducial pins, or needles can itself distort the target anatomy and reduce the repeatability of the evaluation [5,60]. By contrast, the present protocol enables a non-contact, reproducible validation approach based on publicly available medical datasets and virtual measurements within MxR [39]. Users acquire spatial data using virtual probes in the tracked space of an HMD, enabling accurate and repeatable measurements without physically contacting the model or patient [23,25]. This approach is non-destructive, can be performed from arbitrary viewpoints, and supports an unlimited number of repetitions. It therefore combines medical relevance with high reproducibility and efficiency.

Differences in study objectives have also led to inconsistent definitions of "accuracy" in prior MRN research. Reported metrics vary widely—from surface projection overlaps and cranial suture offsets to qualitative and quantitative comparisons with commercial navigation systems [6,60-62]. Many of these measurements are user-dependent and reflect both system and operator performance. While MxR visualization is indeed designed to enhance user perception and bridge skill gaps, system-level accuracy should ideally be assessed through simplified, standardized experiments executed by experienced users [23,25,62]. The present protocol introduces a unified accuracy framework that distinguishes between interpolation and extrapolation domains and explicitly defines metrics for virtual-to-physical and virtual-to-perceptual mappings [39]. This structured taxonomy enables comparable, reproducible accuracy evaluation across different MRN systems and study designs.
